# Synthetic Studies on the Incorporation of *N*-Acetylallosamine in Hyaluronic Acid-Inspired Thiodisaccharides

**DOI:** 10.3390/molecules26010180

**Published:** 2021-01-01

**Authors:** Alejandro E. Cristófalo, María Laura Uhrig

**Affiliations:** 1 Departamento de Química Orgánica, Facultad de Ciencias Exactas y Naturales, Universidad de Buenos Aires, Intendente Güiraldes 2160, Buenos Aires C1428EGA, Argentina; acristofalo@qo.fcen.uba.ar; 2 Centro de Investigaciones en Hidratos de Carbono (CIHIDECAR), CONICET-Universidad de Buenos Aires, Buenos Aires C1428EGA, Argentina

**Keywords:** thiodisaccharides, glucuronic acid, *N*-acetylglucosamine, *N*-acetylallosamine, propargylation, glycomimetics

## Abstract

Two approaches for the synthesis of the thiodisaccharide β-S-GlcA(1→3)β-S-AllNAc are described here. The target disaccharide was a C-3 epimer and thio-analogue of the hyaluronic acid repetitive unit, tuned with a thiopropargyl anomeric group for further click conjugation. Thus, we analysed and tested two convenient sequences, combining the two key steps required to introduce the thioglycosidic bonds and consequently reach the target molecule: the S_N_2 substitution of a good leaving group (triflate) present at C-3 of a GlcNAc derivative and the introduction of the anomeric thiopropargyl substituent. The use of a 2-azido precursor showed to be a convenient substrate for the S_N_2 step. Nevertheless, further protecting group manipulation and the introduction of the thiopropargyl anomeric residue were then required. This approach showed to provide access to a variety of thiodisaccharide derivatives as interesting building blocks for the construction of neoglycoconjugates.

## 1. Introduction

There is a large amount of evidence that the construction of thioglycosidic linkages remains a convenient strategy for the development of sugar-based stable enzyme inhibitors and lectin ligands [1,2,3,4]. In fact, the introduction of thioglycosidic bonds in a complex oligosaccharide chain imparts enhanced stability to the whole structure. Considering the relevant role of carbohydrates in a myriad of normal and aberrant physiological processes, the resulting sugar derivatives can be envisaged as potential tools for the development of carbohydrate-based therapies.

Carbohydrate synthetic chemists can contribute substantially to this field. In fact, considerable efforts have been made to develop suitable synthetic methodologies in this respect [2]. Moreover, even classical methods involving S_N_2-mechanisms deserve further exploration, as they have shown to be both high-yielding and stereoselective processes [5,6,7,8]. Improved conditions by using specific solvent mixtures or additives broaden the possibilities for the access to more complex novel structures, which can be considered analogues of natural compounds [8,9].

Besides, lectin recognition of carbohydrates has been demonstrated to be a highly specific process. Nevertheless, the complexity of sugars in terms of their stereochemical diversity, can be seen as a major advantage for the study of these processes, and deserves further exploration and exploitation. For instance, we have recently demonstrated that *N*-acetylallosamine (AllNAc), the C-3 epimer of *N*-acetylglucosamine (GlcNAc), can be efficiently recognised by the wheat germ agglutinin (WGA), a well-known lectin which has traditionally been described as specific towards *N*-acetylglucosamine. [10].

**Figure 1:**
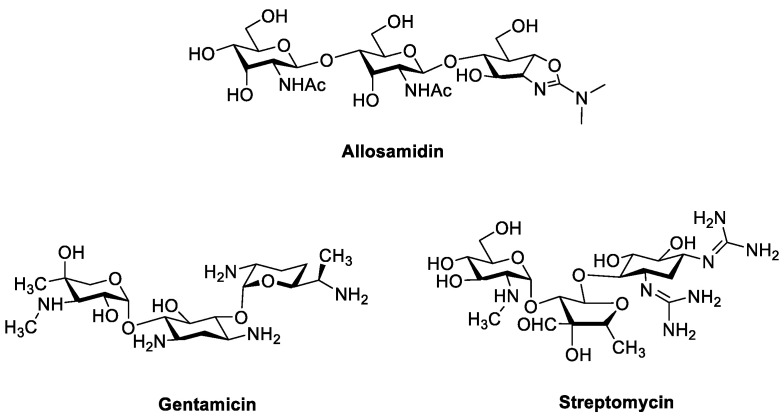
Some examples of relevant rare sugar-based bioactive molecules.

AllNAc is considered as a rare sugar which has only been found in nature as a constituent of allosamidin [11], a metabolite produced by the soil bacteria *Streptomyces* sp., and was the first discovered insect chitinase inhibitor [12,13]. Allosamidin has a pseudotrisaccharide structure with a β-(1→4) disaccharide of AllNAc residues linked to a five-member cyclitol moiety fused to a dimethylaminooxazoline ring (Figure 1). The fused five-member rings mimic the oxazolinium ion intermediate produced during the hydrolysis of insect family-18 chitinases [14]. Interestingly, mammals do not have chitin nor chitin synthase activities, but an acidic mammalian chitinase has been described. Furthermore, this enzyme has been associated to certain asthmatic and allergic conditions [15] and anti-asthmatic activity was reported for allosamidin in a mouse model of asthma [16]. This result reinforces the importance of exploring non-classical carbohydrate structures as candidates for therapeutic purposes, and thus focusing efforts on developing convenient synthetic pathways [17,18]. It should also be mentioned that many antibiotics are composed of deoxy amino monosaccharides and carbasugars, which are considered rare sugars as well. Some examples of these are streptidine and purporosamine residues, present in streptomycin and gentamicin antibiotics, respectively, whose chemical groups and stereochemistry seem to be related to their activity (Figure 1) [19]. In this respect, the presence of rare monosaccharides as constituents of bacterial glycan structures encouraged the development of synthetic inhibitors which could interfere with involved biosynthetic pathways [20].

Taking into consideration the relevant biological roles of GlcNAc and GalNAc as constituents of *N*-linked and *O*-linked oligosaccharides, AllNAc can be considered a promising scaffold for the development of modified carbohydrates. Nevertheless, the reports on the synthesis and properties of AllNAc-containing glycans and their recognition by proteins are scarce [10,21]. To date, as far as we know, only one example of the interaction of AllNAc-containing oligosaccharides with proteins was reported, exhibiting affinity towards sialoadhesins in similar ranges as structurally related GlcNAc-oligosaccharides [22].

On the other hand, glucuronic acid (GlcA) is also considered an interesting building block for the synthesis of glycomimetics. GlcA, together with *N*-acetylhexosamines (GlcNAc and GalNAc) are major constituents of glycosaminoglycans (GAGs), key polysaccharides present in connective tissues and extracellular matrixes [23]. Among GAGs, hyaluronic acid (HA) is characterised by extremely long chains of 10^4^ Da or more, constituted by the non-sulphated repetitive disaccharide [→4)βGlcA(1→3)βGlcNAc(1→] [24,25,26]. Its high prevalence in skin and connective tissues has made it of great interest for both cosmetic industry and osteoarthritis prevention treatments. Remarkably, HA has also been shown to play a role in tumour progression and cancerous conditions, through interactions with proteins such as CD44 [27]. Additionally, HA turnover and the related HA-derived oligosaccharides have also been investigated in this respect [28].

Considering this background, and also encouraged by our latest finding related to the unexpected affinity of AllNAc for WGA [10], we were prone to investigate different synthetic approaches towards new glycomimetic structures constructed from this non-classical sugar building block. Moreover, taking into consideration our work on the synthesis of the hyaluronan mimetic thiodisaccharide, β-*S*-GlcA(1→3)β-*S*-GlcNAc [8], and also the thioglycosylation method developed by us to obtain clickable *N*-acetylhexosamine derivatives [29], we report here two distinct synthetic approaches to obtain the analogous thiodisaccharide β-*S*-GlcA(1→3)β-*S*-AllNAc prepared for further click conjugation. 

## 2. Results and Discussion

**Figure 2:**
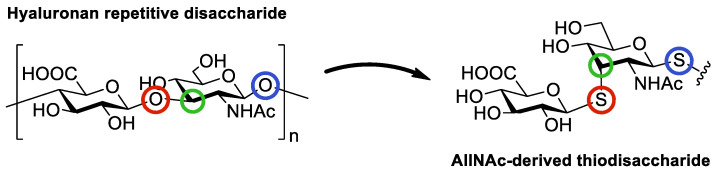
Hyaluronic acid repetitive unit and proposed structural modifications to reach AllNAc-containing thiodisaccharide analogues.

As part of our investigations on the synthesis of hexosamine-containing thiodisaccharides [8] and inspired by the structure of hyaluronic acid, we envisioned the incorporation of AllNAc residues into synthetic disaccharidic thio-analogues. Consequently, key modifications would rather be included by epimerisation of the GlcNAc moiety and transformation of *O*-glycosidic linkages into thioglycosidic bonds to obtain the β-*S*-GlcA(1→3)β-*S*-AllNAc thiodisaccharide (Figure 2).

**Scheme 1:**
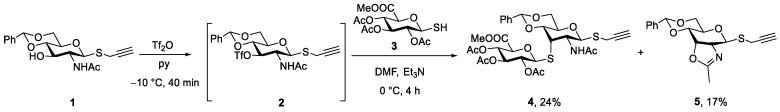
First synthetic approach to the β-*S*-GlcA(1→3)β-*S*-AllNAc thiodisaccharide.

Moreover, the introduction of a thioalkynyl substituent into the anomeric position would allow further click conjugation with azido-scaffolds of different nature, to obtain, for instance, multivalent structures or glycohybrid compounds. Thus, we first explored its introduction in early steps of the synthetic sequence. To achieve this objective, we took advantage of the thio-alkynyl functionalised precursor **1** previously described by us [10], which can be obtained from 2-acetamido-1,3,4,6-tetra-*O*-acetyl-2-deoxy-β-d-glucopyranose in 34% global yield. Using a classic S_N_2 approach, in a 2-step reaction (Scheme 1), we first treated **1** with Tf_2_O/py to obtain the 3-*O*-triflate derivative **2** which was not isolated from the reaction medium [10,30]. The addition of the GlcA-derived thioaldose **3** [9,31,32] and NEt_3_ in anhydrous DMF at 0 °C, led to thiodisaccharide **4**, which could be successfully recovered from the reaction mixture and purified by column chromatography.

As expected, the ^1^H NMR spectrum of **4** showed corresponding signals to the AllNAc and GlcA residues. Regarding the GlcA moiety, diagnostic signals were observed, namely the signal corresponding to the anomeric proton H-1′ as a doublet at 4.68 ppm (*J*_1′,2′_ = 10.2 Hz) and the signal of the methyl ester protons as a singlet at 3.70 ppm. Furthermore, in relation to the hexosamine residue, we could confirm that inversion of the C-3 configuration effectively occurred. The H-3 diagnostic signal appeared at 3.68 ppm, protected due to the effect of the linked sulphur atom, as a triplet with *J*_2,3_ ≅ *J*_3,4_ = 3.9 Hz. This was consistent with the *allo* configuration and thus, a gauche disposition of vicinal H-2 and H-4. Similar values were observed in literature, such as in the case of the β-d-AllNPhth(1→3)-β-d-AllNPhth disaccharide reported by Vasella (H-3′: *δ* 5.54 ppm, t, *J* = 2.8 Hz, H-3: *δ* 4.77 ppm, t, *J* = 3.4 Hz) [21]. 

In the ^13^C NMR spectrum, the signals of both anomeric carbons C-1′ and C-1 appeared at 86.0 and 82.9 ppm, shielded with respect to the oxygenated analogues. Moreover, the signal corresponding to C-3, the position where the substitution occurred, was strongly shielded to 52.4 ppm with respect to the oxygenated C-3 of the precursor **1** (72.9 ppm) [10]. 

Despite the fact that we could isolate the desired product **4**, this reaction proceeded with low yield and led to degradation products together with oxazoline **5**, which could not be avoided even by lowering the reaction temperature to −45 °C. The obtention of compound **5** can be explained by the tendency of GlcNAc 3-*O*-triflates to undergo intramolecular cyclisation [10]. Moreover, this result is one example of the intricate chemistry showed by *N*-acetylhexosamines; the NHAc group in the 2-position is frequently involved in the formation of byproducts such as both 1,2- and 2,3-oxazolines and aziridines [33,34]. Additionally, this group participates in a hydrogen bond network which negatively impacts on reactivity and may hamper glycosylation reactions [35,36]. In this respect, we have described that the use of 2-azido derivatives is a reliable alternative for the construction of (1→3) GlcNAc thiodisaccharides [8]. 

**Scheme 2:**
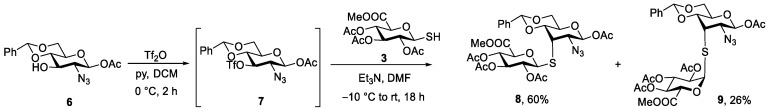
Synthesis of thiodisaccharides **8** and **9**.

Thus, to overcome this drawback, we decided to explore a different synthetic approach. The alternative synthetic pathway to obtain the desired GlcA-AllNAc thiodisaccharide required masking the NHAc group to bypass its interference. Therefore, we chose compound **6** as the starting building block, a 2-azido-glucose derivative which can be obtained from glucosamine hydrochloride in a three-step synthesis previously described by us and others [8,37]. The treatment of **6** with Tf_2_O/py, led to triflyl derivative **7**, which was not isolated. By further treatment with thioaldose **3** and NEt_3_ in anhydrous DMF at −10 °C (Scheme 2), we were pleased to obtain the desired thiodisaccharide **8** in a good 60% yield from **6**. 

In a similar analysis to that of compound **4**, the ^1^H NMR spectrum of **8** could unambiguously confirm the structure of this thiodisaccharide, since the signals corresponding to GlcA and AllN_3_ residues could be identified. The signal of the anomeric proton of the GlcA residue (H-1′) appeared at 5.07 ppm as a doublet (*J*_1′,2′_ = 10.1 Hz), consistent with its β anomeric configuration. Moreover, the doublet at 3.81 ppm corresponded to H-5′ (*J*_4′,5′_ = 9.8 Hz) and the singlet at 3.71 ppm to the methyl ester protons. As for the AllN_3_ residue, the diagnostic H-3 signal appeared at 4.01 ppm as a double doublet with *J*_3,4_ = 3.5, *J*_2,3_ = 4.7 Hz consistent again with its expected *allo* configuration. Furthermore, in the ^13^C NMR spectrum of **8**, the diagnostic signals of C-1′ and C-3 were observed at 84.4 ppm and 46.8 ppm, respectively.

Notably, together with thiodisaccharide **8**, thiodisaccharide **9** was also isolated. Compound **9** resulted from the anomerisation of the GlcA thioaldose, prior to the attack to the GlcN_3_ derivative electrophilic C-3, a process that has been previously proposed to occur through a pyranose ring-opening mechanism [9]. In the ^1^H NMR spectrum of **9**, the signal corresponding to the *S*-GlcA anomeric proton appeared as a deshielded doublet at 5.90 ppm with *J*_1′,2′_ = 5.5 Hz, consistent with an α anomeric configuration. Chemical shift and coupling constant values were also consistent with those described by Kovensky et al. for the α-*S*-GlcA-(1→4)-α-Glc thiodisaccharide (*δ* 5.79 ppm, *J*_1′,2′_ = 5.4 Hz) [9]. The H-5′ signal appeared at 4.84 ppm, remarkably deshielded with respect to the same signal of the β anomer **8** (*δ* 3.81 ppm). Regarding the AllN_3_ residue, the signals corresponding to H-2, H-3, H-4, and H-5 appeared as superimposed multiplets at 3.81–3.71 ppm, clearly shielded with respect to the analogous signals of **8** which appeared at 4.01–3.87 ppm. In the ^13^C NMR spectrum, the signal of the α-*S*-GlcA anomeric carbon (C-1′) appeared at 82.4 ppm, while the signal of C-5′ appeared at 68.2 ppm. Additionally, the diagnostic signal of C-3 holding the sulphur linkage appeared at 46.1 ppm.

Table 1 summarises the characteristic ^1^H and ^13^C-NMR signals of thiodisaccharides **4**, **8** and **9**.

**Scheme 3:**
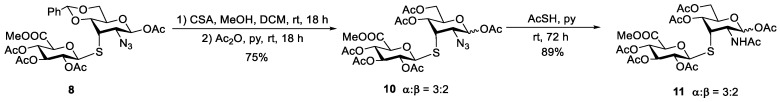
Transformation of thiodisaccharide **8** into **10α**,**β** and **11α**,**β**.

Then, we faced the transformation of thiodisaccharide **8** to achieve the final products. To that aim, we decided to hydrolyse the benzylidene protecting group in the first place to give thiodisaccharide **10α**,**β** and, in a second step, to reincorporate the NHAc functionality, giving the anomeric mixture **11α**,**β** (Scheme 3).

Thus, **10α**,**β** was obtained in 75% yield by treatment of **8** with CSA in MeOH/DCM for 18 h, followed by acetylation. The prolonged acid treatment caused isomerisation of the anomeric functionality (**α/β** 3:2 ratio determined by ^1^H NMR). This consequence was unavoidable, since reducing reaction times led to low conversions. Still, it is noteworthy that this fact was not troublesome because the following reactions could easily be carried out using the anomeric mixture. Thus, to reincorporate the NHAc group, **10α**,**β** was treated with AcSH/py for 72 h at room temperature [38,39] giving **11α,β** as a mixture of anomers (**α**/**β** 3:2 ratio, determined by ^1^H NMR) in 89% global yield. 

**Scheme 4:**
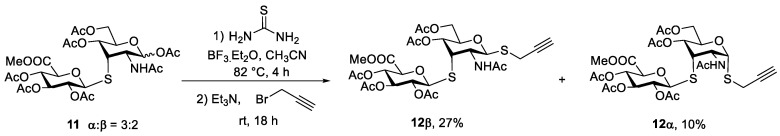
Synthesis of alknyl thiodisaccharides **12β** and **12α** through one-pot thioglycosylation.

The next and final step was the functionalisation of the synthesised thiodisaccharide with a thioalkynyl group to convert it into the originally envisaged clickable product. Moreover, this was an opportunity to extend the one-pot thioglycosylation method developed by us [29]. Concisely, alkynyl thiodisaccharide **12β** was obtained by treating **11α,β**. with thiourea and BF_3_.Et_2_O and further reaction with propargyl bromide and NEt_3_ (Scheme 4). The complex thiodisaccharide **12β** could be obtained in an acceptable 27% yield, considering a two-step transformation. The α-anomer **12α** was also obtained as minor product in 10% yield. Purification of the α- and β-anomers required careful column chromatography using silica gel of <45 μm particle size. Each anomer could be easily characterised by the H-1/H-2 coupling pattern. In the case of **12β**, H-1 appeared at 4.60 ppm, with *J*_1,2_ = 10.2 Hz, due to the transdiaxial disposition of these protons, while in the case of **12α**, H-1 appeared at 5.46 ppm with a *J*_1,2_ = 3.3 Hz, as it is equatorially disposed. These results agree with those previously reported for GlcNAc and GlcNPhth precursors [29]. 

## 3. Materials and Methods

**General Methods.** All solvents were distilled before use. Thin layer chromatography (TLC) was performed on silica gel 60 F254 plates (Merck). The compounds were detected with 5% (*v*/*v*) sulfuric acid in EtOH, containing 0.5% *p*-anisaldehyde. Column chromatography was performed on silica gel 60, particle sizes of 40–63 μm or <45 μm (for thin layer chromatography) from Merck, by elution with the solvents indicated in each case. The ^1^H and ^13^C nuclear magnetic resonance (NMR) spectra were recorded at 25 °C at 500 and 125.7 MHz, respectively, in a Bruker Avance Neo 500 spectrometer. Chemical shifts of ^1^H and ^13^C were reported in parts per million, relative to tetramethylsilane or the residual solvent peak (CHCl_3_: ^1^H: δ 7.26 ppm, ^13^C: δ 77.2 ppm). Assignments of ^1^H and ^13^C were determined by analysis of coupling constants and assisted by 2D ^1^H COSY and 2D ^1^H-^13^C HSQC experiments (see Supplementary Materials). High resolution mass spectra (HRMS) were obtained by electrospray ionisation (ESI) and Q-TOF in a Bruker micrOTOF-Q II spectrometer. Optical rotations were determined in a PerkinElmer 343 polarimeter, at 20 °C in a 1 dm cell. Compound **1** was obtained as previously described [10]. Briefly, this precursor was synthesised from 2-acetamido-1,3,4,6-tetra-*O*-acetyl-2-deoxy-β-d-glucopyranose by one-pot thioglycosylation to introduce the anomeric thioalkynyl group [29] (39% yield), followed by deacetylation (96% yield) and finally protecting 4,6-positions by a classic benzylidene acetal introduction (92% yield). 

**2-Propynyl 4,6-*O*-benzylidene-2-acetamido-2-deoxy-3-*S*-(methyl 2,3,4-tri-*O*-acetyl-β-d-glucopyranosyluronate)-1,3-dithio-β-d-allopyranoside (4).** Compound 1 (70 mg, 0.191 mmol) was dissolved in distilled pyridine (1.0 mL) and the resulting solution was cooled to −10 °C. Then, Tf_2_O (64 μL, 0.382 mmol) was added dropwise and the reaction mixture was stirred under Ar for 40 min. Completion of the reaction was determined by TLC (EtOAc), by observing the consumption of the starting material 1 (R*_f_* = 0.50) and the appearance of a spot corresponding to the product 2 (R*_f_* = 0.92). Then, the mixture was diluted with DCM (3.0 mL) and extracted with HCl 1M (2 × 3.0 mL), NaHCO_3_ s.s. (1 × 3.0 mL) and brine (1 × 3.0 mL). The organic layer was dried over MgSO_4_, filtered, and concentrated under reduced pressure.

The residue was dissolved in DMF (anh., 2.0 mL) and thioaldose **3** (67 mg, 0.191 mmol) was added. The solution was cooled to 0 °C and then Et_3_N (153 μL, 1.1 mmol) was added. The reaction mixture was stirred for 4 h. Then, the mixture was diluted with EtOAc (15 mL) and washed with LiCl 5% (1 × 5.0 mL) and water (1 × 5.0 mL). The organic layer was dried over MgSO_4_, filtered, and concentrated under reduced pressure. Column chromatography of the residue (hexane/EtOAc 7:3 → 1:3) gave first oxazoline **5** (11 mg, 17%) [10] and then thiodisaccharide **4** (31 mg, 24%) [α]_D_^20^ −72,8 (*c* = 1.0, CHCl_3_); ^1^H NMR (500 MHz, CDCl_3_) *δ* 7.49−7.36 (m, 5 H, H-Ar), 6.28 (d, 1 H, *J*_NH,2_ = 9.3 Hz, N*H*), 5.58 (s, 1 H, PhC*H*O), 5.23 (t, 1 H, *J*_2′,3′_ ≅ *J*_3′,4′_ = 9.6 Hz, H-3′), 5.16 (t, 1 H, *J*_3′,4′_ ≅ *J*_4′,5′_ = 9.6 Hz, H-4′), 5.02 (dd, 1 H, *J*_2′,3′_ = 9.6, *J*_1′,2′_ = 10.2 Hz, H-2′), 4.68 (d, 1 H, *J*_1′,2′_ = 10.2 Hz, H-1′), 4.63 (d, 1 H, *J*_1,2_ = 10.5 Hz, H-1), 4.52 (dd, 1 H, *J*_2,3_ = 3.9, J_NH,2_ = 9.3 Hz, *J*_1,2_ = 10.5, H-2), 4.32 (m, 1 H, H-6eq), 3.92 (d, 1 H, *J*_4′,5′_ = 9.6 Hz, H-5′), 3.89 (dd, 1 H, *J*_3,4_ = 3.9, *J*_4,5_ = 8.5 Hz, H-4), 3.76 (m, 2 H, H-5 + H-6ax), 3,70 (s, 3 H, OC*H*_3_), 3.68 (t, 1 H, *J*_2,3_ ≅ *J*_3,4_ = 3.9 Hz, H-3), 3.51, 3.25 (2 dd, 1 H each, *J*_CH,C≡CH_ = 2.5, *J*_CHa,CHb_ = 16.6 Hz, C*H*_2_S), 2.27 (t, 1 H, *J*_CHa,C≡CH_ = *J*_CHb,C≡CH_ = 2.6 Hz, C≡C*H*), 2.01, 2.00, 1.99, 1.83 (4 s, 12 H, 4 × C*H*_3_CO). ^13^C{^1^H} NMR (125.7 MHz, CDCl_3_) *δ* 170.0, 169.9, 169.8, 169.5, 166.8 (*C*O), 137.2, 129.5, 128.5, 126.3 (C-Ar), 101.4 (Ph*C*HO), 86.0 (C-1′), 82.9 (C-1), 79.3 (HC≡*C*), 78.2 (C-4), 75.9 (C-5′), 72.7 (C-3′), 71.9 (H*C*≡C), 70.1 (C-2′), 69.3 (C-4′), 68.9 (C-6), 68.4 (C-5), 53.1 (O*C*H_3_), 52.4 (C-3), 50.2 (C-2), 23.2, 20.7, 20.5 (×2) (*C*H_3_CO), 17.6 (*C*H_2_S). ESI-HRMS: *m/z* [M+Na]^+^ calcd for C_31_H_37_NNaO_13_S_2_, 718.1599; found, 718.1611.

**1-*O*-Acetyl-2-azido-4,6-*O*-benzylidene-2-deoxy-3-*S*-(methyl 2,3,4-tri-*O*-acetyl-β-d-glucopyranosyluronate)-3-thio-β-d-allopyranose (8) and 1-*O*-acetyl-2-azido-4,6-*O*-benzylidene-2-deoxy-3-*S*-(methyl 2,3,4-tri-*O*-acetyl-α-d-glucopyranosyluronate)-3-thio-β-d-allopyranose (9).** Compound 6 (300 mg, 0.894 mmol) was dissolved in DCM (anh., 3.0 mL) and distilled pyridine (1.2 mL). The solution was cooled to 0 °C and Tf_2_O (180 μL, 1.1 mmol) was added dropwise. The reaction mixture was stirred under Ar atmosphere for 2 h. Completion of the reaction was observed by TLC (hexane/EtOAc 1:1) as the spot corresponding to starting material 6 disappeared (R*_f_* = 0.75) and a spot corresponding to the product 7 appeared (R*_f_* = 0.92). The mixture was diluted with EtOAc and extracted with HCl 1M (1 × 10 mL), NaHCO_3_ s.s. (1 × 15 mL) and brine (1 × 15 mL). The organic layer was dried over MgSO_4_, filtered, and concentrated under vacuum.

Crude triflate **7** was dissolved in DMF (anh., 6.6 mL) and thioaldose **3** (1.07 mmol, 375 mg) was added. The mixture was cooled to −10 °C under Ar atmosphere. Then Et_3_N (2.15 mmol, 297 μL) was added and the mixture was allowed to gradually reach room temperature. The reaction mixture was stirred for 18 h until consumption of the starting material (R*_f_* = 0.83, hexane/EtOAc 1:1) and appearance of two spots corresponding to the products (R*_f_* = 0.47 and R*_f_* = 0.53, hexane/EtOAc 1:1) was observed by TLC. Then, the mixture was diluted with EtOAc (25 mL) and washed with LiCl 5% (1 × 10 mL) and water (1 × 10 mL). The organic layer was dried over MgSO_4_, filtered, and concentrated under reduced pressure. Column chromatography of the residue (hexane/EtOAc 9:1 → 1:1) gave first the isomerised product **9** (155 mg, 26%). R*_f_* = 0.53 (hexane/EtOAc 1:1); [α]_D_^20^ +116,1 (*c* 1.0, CHCl_3_). ^1^H NMR (500 MHz, CDCl_3_) *δ* 7.50–7.31 (m, 5 H, H-Ar), 5.96 (d, 1 H, *J*_1,2_ = 8.3 Hz, H-1), 5.90 (d, 1 H, *J*_1′,2′_ = 5.5 Hz, H-1′), 5.53 (s, 1 H, PhC*H*O), 5.46 (t, 1 H, *J*_2′,3′_ ≅ *J*_3′,4′_ ≅ 10.2 Hz H-3′), 5.09 (t, 1 H, *J*_3′,4′_ ≅ *J*_4′,5′_ ≅ 10.2 Hz, H-4′), 5.08 (dd, 1 H, *J*_1′,2′_ = 5.5, *J*_2′,3′_ = 10.2 Hz, H-2′), 4.84 (d, 1 H, *J*_4′,5′_ = 10.2 Hz, H-5′), 4.36 (dd, 1 H, *J*_5,6eq_ = 4,5, *J*_6eq,6ax_ = 10.2 Hz, H-6eq), 3.81–3.71 (m, 4 H, H-2 + H-3 + H-4 + H-5), 3.68 (t, 1 H, *J*_6eq,6ax_ ≅ *J*_5,6ax_ ≅ 10.2 Hz, H-6ax), 3.41 (s, 3 H, OC*H*_3_), 2.19, 2.10, 2.02, 1.97 (4 s, 12 H, 4 × C*H*_3_CO); ^13^C{^1^H} NMR (125.7 MHz, CDCl_3_) *δ* 169.8 (×2), 169.6, 168.8, 168.0 (*C*O), 137.0, 129.2, 128.3, 126.5 (C-Ar), 102.0 (Ph*C*HO), 92.7 (C-1), 82.4 (C-1′), 76.6 (C-4), 70.2 (C-2′), 69.6 (C-3′), 69.3 (C-4′), 68.8 (C-6), 68.2 (C-5′), 67.0 (C-5), 62.2 (C-2), 52.7 (O*C*H_3_), 46.1 (C-3), 21.1, 20.8, (×2), 20.7 (*C*H_3_CO). ESI-HRMS: *m/z* [M+Na]^+^ calcd for C_28_H_33_N_3_NaO_14_S: 690.1575, found: 690.1573.

Then, thiodisaccharide **8** (358 mg, 60%) was eluted from the column. R*_f_* = 0.47 (hexane/EtOAc 1:1); [α]_D_^20^ −65.0 (*c* 0.9, CHCl_3_). ^1^H NMR (500 MHz, CDCl_3_) *δ* 7.53–7.41 (m, 5 H, H-Ar), 5.62 (d, 1 H, *J*_1,2_ = 8.7 Hz, H-1), 5.54 (s, 1 H, PhC*H*O), 5.22 (dd, 1 H, *J*_4′,5′_ = 9.8, *J*_3′,4′_ = 9.5 Hz, H-4′), 5.13 (dd, 1 H, *J*_2′,3′_ = 8.7, *J*_3′,4′_ = 9.5 Hz, H-3′), 5.07 (d, 1 H, *J*_1′,2′_ = 10.1 Hz, H-1′), 5.03 (dd, 1 H, *J*_2′,3′_ = 8.7, *J*_1′,2′_ = 10.1 Hz, H-2′), 4.34 (dd, 1 H, *J*_5,6eq_ = 5.2, *J*_6eq,6ax_ = 10.3 Hz, H-6eq), 4.15 (ddd, 1 H, *J*_5,6eq_ = 5.2, *J*_4,5_ = 9.2, *J*_5,6ax_ = 10.3 Hz, H-5), 4.01 (dd, 1 H, *J*_3,4_ = 3.5, *J*_2,3_ = 4.7 Hz, H-3), 3.94 (dd, 1 H, *J*_2,3_ = 4.7, *J*_1,2_ = 8.7 Hz, H-2), 3.87 (dd, 1 H, *J*_3,4_ = 3.5, *J*_4,5_ = 9.2 Hz, H-4), 3.81 (d, 1 H, *J*_4′,5′_ = 9.8 Hz, H-5′), 3.74 (t, 1 H, *J*_6eq,6ax_ ≅ *J*_5,6ax_ ≅ 10.3 Hz, H-6ax), 3.71 (s, 3 H, OC*H*_3_), 2.15, 2.01, 2.00, 1.97 (4 s, 12 H, 4 × C*H*_3_CO); ^13^C{^1^H} NMR (125.7 MHz, CDCl_3_) *δ* 170.1, 169.4, 169.3, 168.5, 166.8 (*C*O), 136.9, 129.9, 128.7, 126.3 (C-Ar), 102.3 (Ph*C*HO), 92.1 (C-1), 84.4 (C-1′), 78.8 (C-4), 76.3 (C-5′), 73.2 (C-3′), 70.3 (C-2′), 69.2 (C-4′), 68.6 (C-6), 65.3 (C-5), 60.8 (C-2), 53.0 (O*C*H_3_), 46.8 (C-3), 21.0, 20.7 (×2), 20.6 (*C*H_3_CO). ESI-HRMS: *m/z* [M+Na]^+^ calcd for C_28_H_33_N_3_NaO_14_S: 690.1575, found: 690.1543.

**1,4,6-tri-*O*-Acetyl-2-azido-2-deoxy-3-*S*-(methyl 2,3,4-tri-*O*-acetyl-β-d-glucopyranosyluronate)-3-thio-α,β-d-allopyranoside (10α,β).** Compound 8 (350 mg, 0.52 mmol) was treated with CSA (219 mg, 0.94 mmol) in a MeOH/DCM 1:1 (44 mL) solution and stirred for 18 h at room temperature. Then, the reaction was quenched by adding Et_3_N and the mixture was concentrated under vacuum. The residue was dissolved in pyridine (10 mL) and treated with Ac_2_O (10 mL, 0.1 mol) for 18 h at room temperature. After this, MeOH was added and the solvents were evaporated under vacuum. Column chromatography (hexane/EtOAc 4:1 → 1:1) gave 10α,β (260 mg, 75%) as an anomeric mixture in an α/β 3:2 relationship determined by ^1^H NMR, R*_f_* = 0.38 (hexane/EtOAc 1:1). ^1^H NMR (500 MHz, CDCl_3_) *δ* 6.11 (s, 1.65 H, H-1α), 5.76 (d, 1.00 H, *J*_1,2_ = 6.8 Hz, H-1β), 5.38 (ddd, 1.65 H, *J*_5,6a_ = 3.3 *J*_5,6b_ = 4.4, *J*_4,5_ = 7.3 Hz, H-5α), 5.32–5.21 (m, 5.30 H, H-3′α + H-4′α + H-3′β + H-4′β), 5.12 (dd, 1.65 H, *J*_2′,3′_ = 9.4 *J*_1′,2′_ = 10.0 Hz H-2′α), 5.07 (dd, 1.65 H, *J*_2′,3′_ = 9.1 *J*_1′,2′_ = 9.9 Hz H-2′β), 4.98 (dd, 1.00 H, *J*_3,4_ = 3.9, *J*_4,5_ = 7.2 Hz, H-4β), 4.67 (d, 1.65 H, *J*_1′,2′_ = 10.0 Hz, H-1′α), 4.55 (d, 1.00 H, *J*_1′,2′_ = 9.9 Hz, H-1′β), 4.44 (dd, 1.65 H, *J*_5,6a_ = 3.3, *J*_6a,6b_ = 12.2 Hz, H-6aα), 4.29 (dd, 1.00 H, *J*_5,6a_ = 4.1, *J*_6a,6b_ = 12.2 Hz, H-6aβ), 4.25–4.21 (m, 2.65 H, H-2α + H-6bβ), 4.14–4.05 (m, 5.95 H, H-4α + H-5′α + H-6bα + H-5β), 4.01 (d, 1.00 H, *J*_4′,5′_ = 9.7 Hz, H-5′β), 3.97 (t, 1.00 H, *J*_2,3_ ≅ *J*_3,4_ = 4.2 Hz, H-3β), 3.82 (dd, 1.00 H, *J*_2,3_ = 4.2, *J*_1,2_ = 6.8 Hz, H-2β), 3.77–3.72 (m, 9.60 H, COOC*H_3_*-α + COOC*H_3_*-β + H-3α), 2.17–2.01 (m, 47.7 H, 6 C*H*_3_CO-α + 6 C*H*_3_CO-β); ^3^C{^1^H} NMR (125.7 MHz, CDCl_3_) *δ* 170.9 (×2), 170.2 (×2), 170.1, 169.9, 169.7, 169.4 (×2), 168.7, 166.6, 166.5 (*C*O), 99.4 (C-1α), 92.1 (C-1β), 83.5 (C-1′β), 83.1 (C-1′α), 82.7 (C-4α), 76.6 (C-5′β), 76.1 (C-5′α), 73.2 (C-5β), 73.1 (C-3′α + C-3′β), 71.2 (C-5α), 70.2 (C-2′β), 69.8 (C-2′α), 69.1 (C-4′β), 69.0 (C-2α + C-4′α), 67.7 (C-4β), 62.5 (C-6α), 62.4 (C-6β), 60.3 (C-2β), 53.1 (COO*C*H*_3_*-α + COO*C*H*_3_*-β), 46.4 (C-3α), 44.8 (C-3β), 21.3, 21.2, 21.1, 21.0, 20.9 (×3), 20.8 (×2), 20.7 (×2), 20.6 (*C*H_3_COα + *C*H_3_COβ). ESI-HRMS: *m/z* [M+Na]^+^ calcd for C_25_H_33_N_3_NaO_16_S: 686.1474, found: 686.1463.

**1,4,6-tri-*O*-Acetyl-2-acetamido-2-deoxy-3-*S*-(methyl 2,3,4-tri-*O*-acetyl-β-d-glucopyranosyluronate)-3-thio-α,β-d-allopyranoside (11α,β).** The anomeric mixture 10α,β (250 mg, 0.38 mmol) was dissolved in pyridine (7.5 mL) and thioacetic acid (570 µL, 7.5 mmol) was added under Ar atmosphere. The reaction mixture was stirred for 72 h at room temperature. Then, solvents were evaporated under reduced pressure and column chromatography of the residue (hexane/EtOAc 1:1 → 1:9) gave the product 11α,β (227 mg, 89%) as an anomeric mixture in an α/β 3:2 relationship determined by ^1^H NMR, R*_f_* = 0.51 (EtOAc). ^1^H NMR (500 MHz, CDCl_3_) *δ* 6.31 (d, 1.0 H, *J*_NH,2_ = 9.5 Hz, N*H*-β), 6.21 (s, 1.5 H, H-1α), 5.93 (d, 1.5 H, *J*_N*H*,2_ = 5.4 Hz, N*H-*α), 5.61 (d, 1.0 H, *J*_1,2_ = 6.9 Hz, H-1β), 5.28–5.20 (m, 6.5 H, H-5α + H-3′α + H-4′α + H-3′β + H-4′β), 5.08 (t, 1.0 H, *J*_1′,2′_ ≅ *J*_2′,3′_ = 10.0 Hz, H-2′β), 5.05 (t, 1.5 H, *J*_1′,2′_ ≅ *J*_2′,3′_ = 10.0 Hz, H-2′α), 4.98 (dd, 1.0 H, *J*_3,4_ = 4.0 Hz, *J*_4,5_ = 7.6 Hz H-4β), 4.64 (d, 1.5 H, *J*_1′,2′_ = 10.0 Hz, H-1′α), 4.47 (ddd, 1.0 H, *J*_NH,2_ = 9.5, *J*_1,2_ = 6.9, *J*_2,3_ = 4.0 Hz, H-2β), 4.44 (dd, 1.5 H, *J*_5,6a_ = 3.1, *J*_6a,6b_ = 12.2 Hz, H-6aα), 4.40 (d, 1.0 H, *J*_1′,2′_ = 10.0 Hz, H-1′β), 4.36 (dd, 1.0 H, *J*_5,6a_ = 4.3, *J*_6a,6b_ = 12.1 Hz, H-6aβ), 4.29 (t, 1.5 H, *J*_NH,2_ ≅ *J*_2,3_ = 5.4 Hz, H-2α), 4.17 (dd, 1.0 H, *J*_5,6b_ = 4.6, *J*_6a,6b_ = 12.1 Hz, H-6bβ), 4.06–4.00 (m, 4.5 H, H-4α + H-6bα + H-5′α), 4.00–3.95 (m, 2.0 H, H-5β + H-5′β), 3.90 (t, 1.0 H, *J*_2,3_ ≅ *J*_3,4_ = 4.0 Hz, H-3β), 3.84 (dd, 1.5 H, *J*_2,3_ = 5.4, *J*_3,4_ = 8.9 Hz, H-3α), 3.73 (s, 4.5 H, COOC*H_3_*-α), 3.72 (s, 3.0 H, COOC*H_3_*-β), 2.13, 2.12, 2.11, 2.10, 2.09, 2.08, 2.07, 2.06, 2.04, 2.03, 2.02 (×2), 1.98 (11 s, 45 H, 6 × C*H*_3_CO-α + 6 × C*H*_3_CO-β); ^13^C{^1^H} NMR (125.7 MHz, CDCl_3_) *δ* 171.3, 171.0, 170.8 (×2), 170.2, 170.1 (×2), 170.0, 169.5 (×3), 169.4, 169.3, 169.2, 166.8, 166.5 (*C*O), 99.8 (C-1α), 92.4 (C-1β), 84.8 (C-1′β), 84.1 (C-1′α), 81.9 (C-4α), 76.2 (C-5′β), 76.0 (C-5′α), 72.9 (×2), 72.7, 72.6 (C-5α + C-5β + C-3′α + C-3′β), 70.1 (C-2′α), 69.9 (C-2′β), 69.2 (C-4′β), 69.0 (C-4′α), 68.4 (C-4β), 62.9 (C-6α), 62.3 (C-6β), 57.8 (C-2α), 53.2 (COO*C*H*_3_*-α), 53.1 (COO*C*H*_3_*-β), 49.2 (C-2β), 47.4 (C-3α), 47.0 (C-3β), 23.3, 23.2, 21.3, 21.2 (×2), 21.1, 21.0, 20.9, 20.8 (×2), 20.7 (×2), 20.6 (×2) (*C*H_3_COα + *C*H_3_COβ). ESI-HRMS): *m/z* [M+Na]^+^ calcd for C_27_H_37_NNaO_17_S: 702.1674, found: 702.1653.

**2-Propynyl 4,6-di-*O*-acetyl-2-acetamido-2-deoxy-3-*S*-(methyl 2,3,4-tri-*O*-acetyl-β-d-glucopyranosyluronate)-1,3-dithio-β-d-allopyranoside (12β) and 2-propynyl 4,6-di-*O*-acetyl-2-acetamido-2-deoxy-3-*S*-(methyl 2,3,4-tri-*O*-acetyl-β-d-glucopyranosyluronate)-1,3-dithio-α-d-allopyranoside (12α).** The synthesis was achieved by performing our previously described one-pot thioglycosylation method, starting from the anomeric mixture 11α,β (217 mg, 0.32 mmol), thiourea (190 mg, 1.44 mmol) and BF_3_.Et_2_O (175 μL, 0.80 mmol) in CH_3_CN (anh., 2.0 mL). The reaction mixture was heated at 82 °C under Ar atmosphere until disappearance of the starting material 11α,β (R*_f_* = 0.72) and appearance of a spot in the origin, which was ascribed to the intermediate isothiouronium salt, and was observed by TLC (EtOAc) (4 h). Then, NEt_3_ (0.31 mL, 1.28 mmol) and propargyl bromide (68 μL, 0.35 mmol) were added and the reaction mixture was stirred at room temperature for 18 h. TLC analysis (EtOAc) revealed two spots corresponding to the product 12β (R*_f_* = 0.83) and its α anomer 12α (R*_f_* = 0.81). The mixture was concentrated under vacuum and the residue was dissolved in EtOAc (25 mL) and washed with water (1 × 10 mL). The organic layer was dried (MgSO_4_), filtered and concentrated under vacuum. Column chromatography (silica gel of <45 μm particle size, CHCl_3_/MeOH 100:0 → 97:3) afforded first compound 12β (60 mg, 27%). R*_f_* = 0.83 (EtOAc); [α]_D_^20^ −27.0 (*c* 0.5, CHCl_3_). ^1^H NMR (500 MHz, CDCl_3_) *δ* 6.27 (d, 1 H, *J*_N*H*,2_ = 9.2 Hz, N*H*), 5.25 (t, 1 H, *J*_2′,3′_ ≅ *J*_3′,4′_ = 9.3 Hz, H-3′), 5.16 (dd, 1 H, *J*_3′,4′_ = 9.3, *J*_4′,5′_ = 9.8 Hz, H-4′), 5.01 (dd, 1 H, *J*_2′,3′_ = 9.3, *J*_1′,2′_ = 10.0 Hz, H-2′), 4.91 (dd, 1 H, *J*_3,4_ = 3.9, *J*_4,5_ = 10.1 Hz, H-4), 4.60 (d, 1 H, *J*_1,2_ = 10.2 Hz, H-1), 4.52 (ddd, 1 H, *J*_2,3_ = 3.9, *J*_N*H*,2_ = 9.2, *J*_1,2_ = 10.2 Hz, H-2), 4.37 (d, 1 H, *J*_1′,2′_ = 10.0 Hz, H-1′), 4.26–4.19 (m, 2 H, H-6a + H-6b), 3.97 (d, 1 H, *J*_4′,5′_ = 9.8 Hz, H-5′), 3.82 (t, 1 H, *J*_2,3_ ≅ *J*_3,4_ = 3.9 Hz, H-3), 3.77–3.69 (m, 4 H, OC*H*_3_ + H-5), 3.52, 3.24 (2 dd, 1 H each, *J*_CH2,C≡CH_ = 2.6, *J*_CH2Sgem_ = 16.6 Hz, C*H*_2_S), 2.25 (t, 1 H, *J*_CH2,C≡CH_ = 2.6 Hz, C≡C*H*), 2.12, 2.09, 2.08, 2.03, 2.02, 1.98 (6 s, 18 H, 6 × C*H*_3_CO); ^13^C{^1^H} NMR (125.7 MHz, CDCl_3_) *δ* 170.9, 170.1, 170.0, 169.7, 169.5, 169.4, 166.7 (*C*O), 86.0 (C-1′), 82.9 (C-1), 79.3 (*C*≡CH), 76.0 (C-5′), 73.9 (C-5), 72.6 (C-3′), 71.8 (C≡*C*H), 70.4 (C-2′), 69.3 (C-4′), 68.6 (C-4), 62.4 (C-6), 53.3 (C-3), 53.1 (O*C*H_3_), 49.7 (C-2), 23.2, 21.0, 20.9 (×2), 20.7, 20.6 (*C*H_3_CO), 17.5 (*C*H_2_S). ESI-HRMS: *m/z* [M+Na]^+^ calcd for C_28_H_37_NNaO_15_S_2_: 714.1497, found: 714.1485.

The α anomer **12α** was eluted next from the column (23 mg, 10%). R*_f_* = 0.81 (EtOAc); [α]_D_^20^ −37.7 (*c* 0.4, CHCl_3_). ^1^H NMR (500 MHz, CDCl_3_) *δ* 6.14 (d, 1 H, *J*_N*H*,2_ = 5.8 Hz, N*H*), 5.46 (d, 1 H, *J*_1,2_ = 3.3 Hz, H-1), 5.36 (ddd, 1 H, *J*_6a,5_ = 3.4, *J*_6b,5_ = 5.4, *J*_4,5_ = 6.6 Hz, H-5), 5.26 (t, 1 H, *J*_2′,3′_ ≅ *J*_3′,4′_ = 9.3 Hz, H-3′) 5.20 (dd, 1 H, *J*_3′,4′_ = 9.2, *J*_4′,5′_ = 9.8 Hz, H-4′), 5.03 (dd, 1 H, *J*_2′,3′_ = 9.3, *J*_1′,2′_ = 10.0 Hz, H-2′), 4.64 (d, 1 H, *J*_1′,2′_ = 10.0 Hz, H-1′), 4.43 (dd, 1 H, *J*_6a,5_ = 3.4, *J*_6a,6b_ = 12.1 Hz, H-6a), 4.37 (ddd, 1 H, *J*_1,2_ = 3.3, *J*_N*H*,2_ = 5.8, *J*_2,3_ = 6.6, Hz, H-2), 4.17 (dd, 1 H, *J*_6b,5_ = 5.4, *J*_6a,6b_ = 12.1 Hz, H-6b), 4.09 (dd, 1 H, *J*_4,5_ = 6.6, *J*_3,4_ = 8.0 Hz, H-4), 4.02 (d, 1 H, *J*_4′,5′_ = 9.8 Hz, H-5′), 3.87 (dd, 1 H, *J*_2,3_ = 6.6, *J*_3,4_ = 8.0 Hz, H-3), 3.73 (s, 3 H, OC*H*_3_), 3.49, 3.34 (2 dd, 1 H each, *J*_CH2,C≡CH_ = 2.6, *J*_CH2Sgem_ = 16.7 Hz, C*H*_2_S), 2.26 (t, 1 H, *J*_CH2,C≡CH_ = 2.6 Hz, C≡C*H*), 2.11, 2.08 (×2), 2.02 (×3) (6 s, 18 H, 6 × C*H*_3_CO); ^13^C{^1^H} NMR (125.7 MHz, CDCl_3_) *δ* 170.8, 170.7, 170.2, 170.0, 169.5 (×2), 166.6 (*C*O), 87.9 (C-1), 84.4 (C-1′), 82.0 (C-4), 79.4 (*C*≡CH), 75.9 (C-5′), 72.9 (C-3′), 73.0 (C-3′), 72.2 (C≡*C*H), 71.9 (C-5), 70.4 (C-2′), 69.0 (C-4′), 62.9 (C-6), 58.4 (C-2), 53.2 (O*C*H_3_), 49.3 (C-3), 23.3, 21.2, 20.9, 20.8, 20.7, 20.6 (*C*H_3_CO), 18.6 (*C*H_2_S). ESI-HRMS: *m/z* [M+Na]^+^ calcd for C_28_H_37_NNaO_15_S_2_: 714.1497, found: 714.1492. 

## 4. Conclusions

Herein, we described different synthetic approaches to obtain the thiodisaccharide β-*S*-GlcA(1→3)β-*S*-AllNAc, functionalised with a thiopropargyl anomeric substituent, to make it suitable for click conjugation. Thus, we studied two distinct sequences involving the two key steps required for the introduction of both structural features of this thiodisaccharide, namely, the anomeric thiopropargylation of the GlcNAc residue and the (1→3) thioglycosidic bond formation.

The first approach exploited an already thiopropargylated β-GlcNAc derivative, which was easily obtained in our laboratory from glucosamine pentaacetate by a one-pot procedure [10,29], and involved an S_N_2 displacement of a triflate group located at C-3, by a β-GlcA derived thioaldose. Although the yield was unfortunately low because the participation of the acetamido group led to undesired secondary products, this short sequence readily led to the envisaged final product **4**, in an 8.3% global yield from glucosamine pentaacetate. It should be mentioned that this yield comprises both thioglycosylation steps.

Nevertheless, in an attempt to improve this yield, we planned to perform first the construction of the (1→3) thioglycosydic bond and then, the introduction of the thiopropargyl anomeric group in a second instance. In this approach, the acetamido group had to be avoided, and thus, we started from 2-azidoglucose, a commercial and easily obtained known precursor frequently used in glycosylation reactions. Thus, the S_N_2 reaction between the β-GlcA derived thioaldose and the corresponding 4,6-*O*-benzylidene-3-*O*-triflate-2-azido-Glc derivative **7**, successfully led to thiodisaccharide **8**. Interestingly, the anomerised thiodisaccharide **9** was also isolated, in accordance with previous reported results on the construction of GlcA thiodisaccharides. Further transformation of the azido group by reaction with thioacetic acid, followed by thiopropargylation, led to the pursued product **12β**, together with a minor amount of its anomer **12α**. The global yield of **12β** from precursor **6** was 10.8% in this alternative sequence.

Compounds **4** and **12β** share the same thiodisaccharide skeleton, differing only in the protecting groups of the hexosamine moieties. Considering the complex structure of **4**, the straightforward synthetic pathway depicted first, and the high availability of the precursors needed, the overall 8.3% yield reported here remains quite acceptable. The second assayed sequence, in turn, requires the more complex precursor **6** as starting material which is, however, easily available through standard carbohydrate synthetic methods. Although the global yield was not substantially improved—both key thioglycosylation steps remained challenging no matter the sequence order—this second pathway brought about an interesting family of thiodisaccharide derivatives, which were purified and fully characterised. Moreover, evidence is provided here that the one-pot BF_3_OEt_2_/SC(NH_2_)_2_/propargylbromide-mediated thioglycosylation reaction previously described [29], can be extended to more complex structures.

Indeed, all the products described here can be considered as useful building blocks for further synthesis of a wide range of more complex neoglycoconjugates and glycomimetics through glycosylation, thioglycosylation or copper(I)-catalysed alkyne–azide cycloaddition (CuAAC) reactions. As stated before, the availability of conveniently protected structures, even more, containing rare sugar residues is important to fulfil these objectives.

The introduction of robust thioglycosidic linkages is not only a synthetic challenge for organic chemists, but also pursues the access to glycomimetic derivatives with improved resistance to hydrolytic processes which are of great interest in glycobiology. In this respect, this study contributes to the understanding of thioglycosylation reactions to obtain some of such glycomimetics, particularly having challenging sugar moieties as hexosamines and uronic acid residues. Further studies on the click conjugation of these thiodisaccharides onto azido-scaffolds are in progress.

## Figures and Tables

**Table 1:** ^1^H and ^13^C-NMR characteristic signals of thiodisaccharides **4**, **8** and **9**. ^1^

	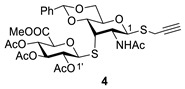	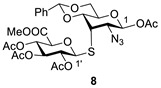	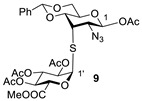
H-1	4.63 (*J*_1,2_ = 10.5)	5.62 (*J*_1,2_ = 8.7)	5.96 (*J*_1,2_ = 8.3)
H-2	4.52 (*J*_2,3_ = 3.9)	3.94 (*J*_2,3_ = 4.7)	3.81–3.71 ^2^
H-3	3.68 (*J*_3,4_ = 3.9)	4.01 (*J*_3,4_ = 3.5)	3.81–3.71 ^2^
H-4	3.89 (*J*_4,5_ = 8.5)	3.87 (*J*_4,5_ = 9.2)	3.81–3.71 ^2^
H-5	3.76 ^2^	4.15 (*J*_5,6eq_ = 5.2, *J*_5,6ax_ = 10.3)	3.81–3.71 ^2^
H-6ax	3.76 ^2^	3.74 (*J*_6eq,6ax_ ≅ 10.3)	3.68 (*J*_6eq,6ax_ ≅ *J*_5,6ax_ ≅ 10.2)
H-6eq	4.32 ^2^	4.34	4.36 (*J*_5,6eq_ = 4,5)
H-1′	4.68 (*J*_1′,2′_ = 10.2)	5.07 (*J*_1′,2′_ = 10.1)	5.90 (*J*_1′,2′_ = 5.5)
H-2′	5.02 (*J*_2′,3′_ = 9.6)	5.03 (*J*_2′,3′_ = 8.7)	5.08 (*J*_2′,3′_ = 10.2)
H-3′	5.23 (*J*_3′,4′_ = 9.6)	5.13 (*J*_3′,4′_ = 9.5)	5.46 (*J*_3′,4′_ ≅ 10.2)
H-4′	5.16 (*J*_4′,5′_ = 9.6)	5.22 (*J*_4′,5′_ = 9.8)	5.09 (*J*_4′,5′_ ≅ 10.2)
H-5′	3.92	3.81	4.84
C-1	82.9	92.1	92.7
C-3	52.4	46.8	46.1
C-1′	86.0	84.4	82.4
C-5′	75.9	76.3	68.2

^1^ Chemical shifts and coupling constants (*J*) are expressed in ppm and Hz, respectively. ^2^ Superposition of the signals precluded the determination of the coupling constants.

## Data Availability

Data sharing not applicable.

## Supplementary Materials

Copies of 1D and 2D NMR spectra of all compounds are available online.

## Sample Availability

Samples of the compounds **4**, **8**, **9**, **10α**,**β**, **11α**,**β**, **12****β** and **12****α** are available from the authors.
