# Supplementary material for: Synthetic Studies on the Incorporation of N-Acetylallosamine in Hyaluronic Acid-Inspired Thiodisaccharides
Source: Molecules. 2021 Jan 1;26(1):180. doi: 10.3390/molecules26010180 (PMC7796257; doi:10.3390/molecules26010180)
Supplement: Supplementary file 1 [file molecules-26-00180-s001.pdf]

Synthetic studies on the incorporation of N-acetylallosamine in hyaluronic acid inspired thiodisaccharides

Alejandro E. Cristófalo[a,b] and María Laura Uhrig[a,b]*

[a] Universidad de Buenos Aires. Facultad de Ciencias Exactas y Naturales. Departamento de Química Orgánica, Intendente Güiraldes 2160 (C1428EHA), Buenos Aires, Argentina.
[b] CONICET- Universidad de Buenos Aires, Centro de Investigaciones en Hidratos de Carbono (CIHIDECAR), Buenos Aires, Argentina.

* Correspondance: María Laura Uhrig, e-mail: mluhrig@qo.fcen.uba.ar; phone: +54 011 528 58535, ORCID: https://orcid.org/0000-0002-6980-4141.

Academic Editors: Patrick M. Martin and Sandrine Bouquillon

Keywords: Thiodisaccharides; Glucuronic acid; N-Acetylglucosamine; N-Acetylallosamine; Propargylation; Glycomimetics.

Table of Contents

^{1}H NMR spectrum of **4**	S2
^{13}C NMR spectrum of **4**	S3
^{1}H-^{1}H COSY NMR spectrum of **4**	S4
^{1}H-^{13}C HSQC NMR spectrum of **4**	S5
^{1}H NMR spectrum of **8**	S6
^{13}C NMR spectrum of **8**	S7
^{1}H-^{1}H COSY NMR spectrum of **8**	S8
^{1}H-^{13}C HSQC NMR spectrum of **8**	S9
^{1}H NMR spectrum of **9**	S10
^{13}C NMR spectrum of **9**	S11
^{1}H-^{1}H COSY NMR spectrum of **9**	S12
^{1}H-^{13}C HSQC NMR spectrum of **9**	S13
^{1}H NMR spectrum of **10α,β**	S14
^{13}C NMR spectrum of **10α,β**	S15
^{1}H NMR spectrum of **11α,β**	S16
^{13}C NMR spectrum of **11α,β**	S17
^{1}H NMR spectrum of **12α**	S18
^{13}C NMR spectrum of **12α**	S19
^{1}H-^{1}H COSY NMR spectrum of **12α**	S20
^{1}H-^{13}C HSQC NMR spectrum of **12α**	S21
^{1}H NMR spectrum of **12β**	S22
^{13}C NMR spectrum of **12β**	S23
^{1}H-^{1}H COSY NMR spectrum of **12β**	S24
^{1}H-^{13}C HSQC NMR spectrum of **12β**	S25

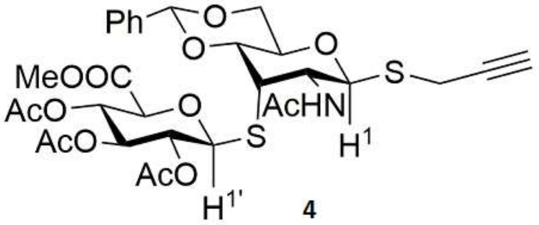
Ph
O
O
O
MeOOC
AcO
AcO
AcO
O
S
AcHN
S
H1
H1'

4

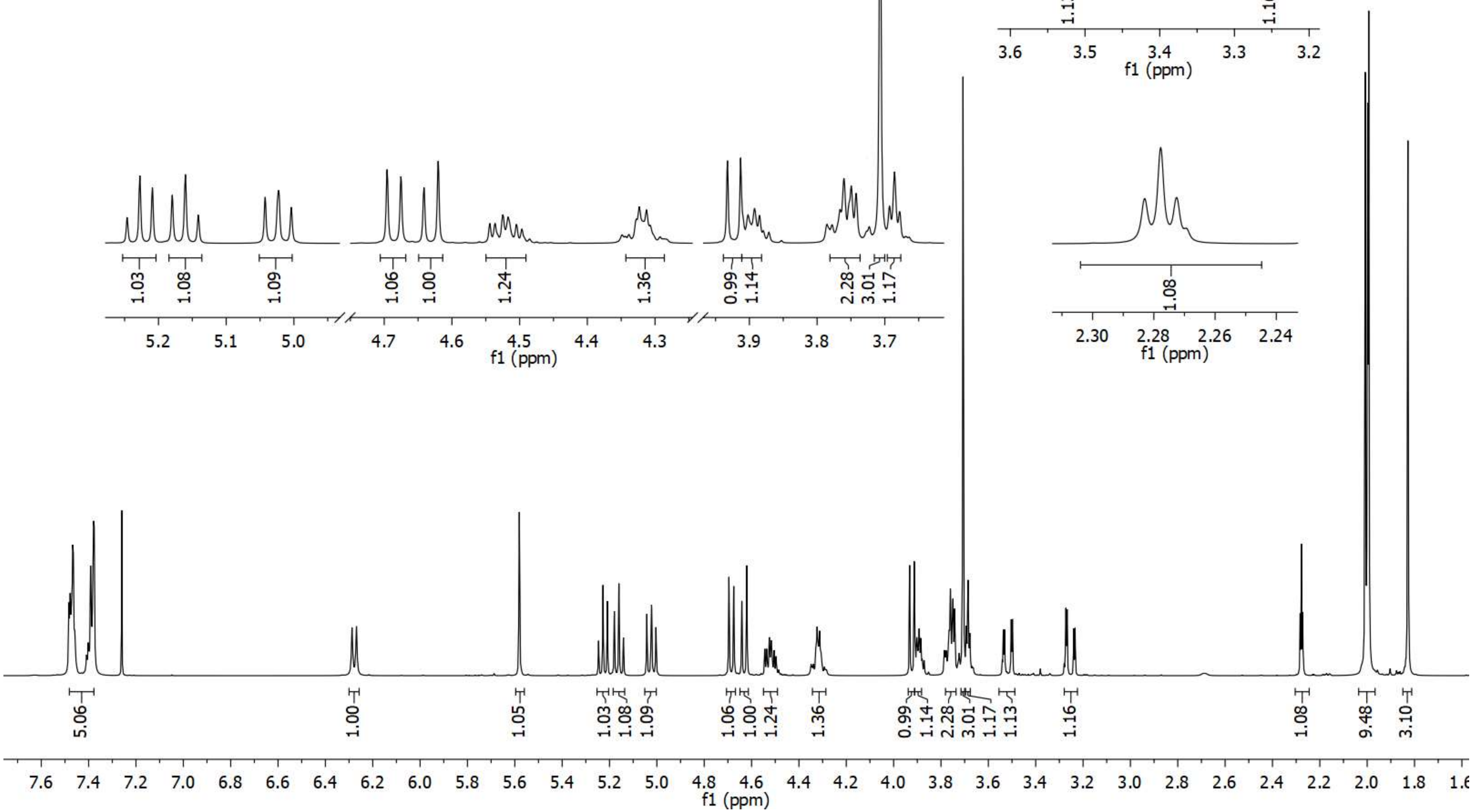
f1 (ppm)
5.06
1.00
1.05
1.03
1.08
1.09
1.06
1.00
1.24
1.36
0.99
1.14
2.28
3.01
1.17
1.13
1.16
1.08
9.48
3.10

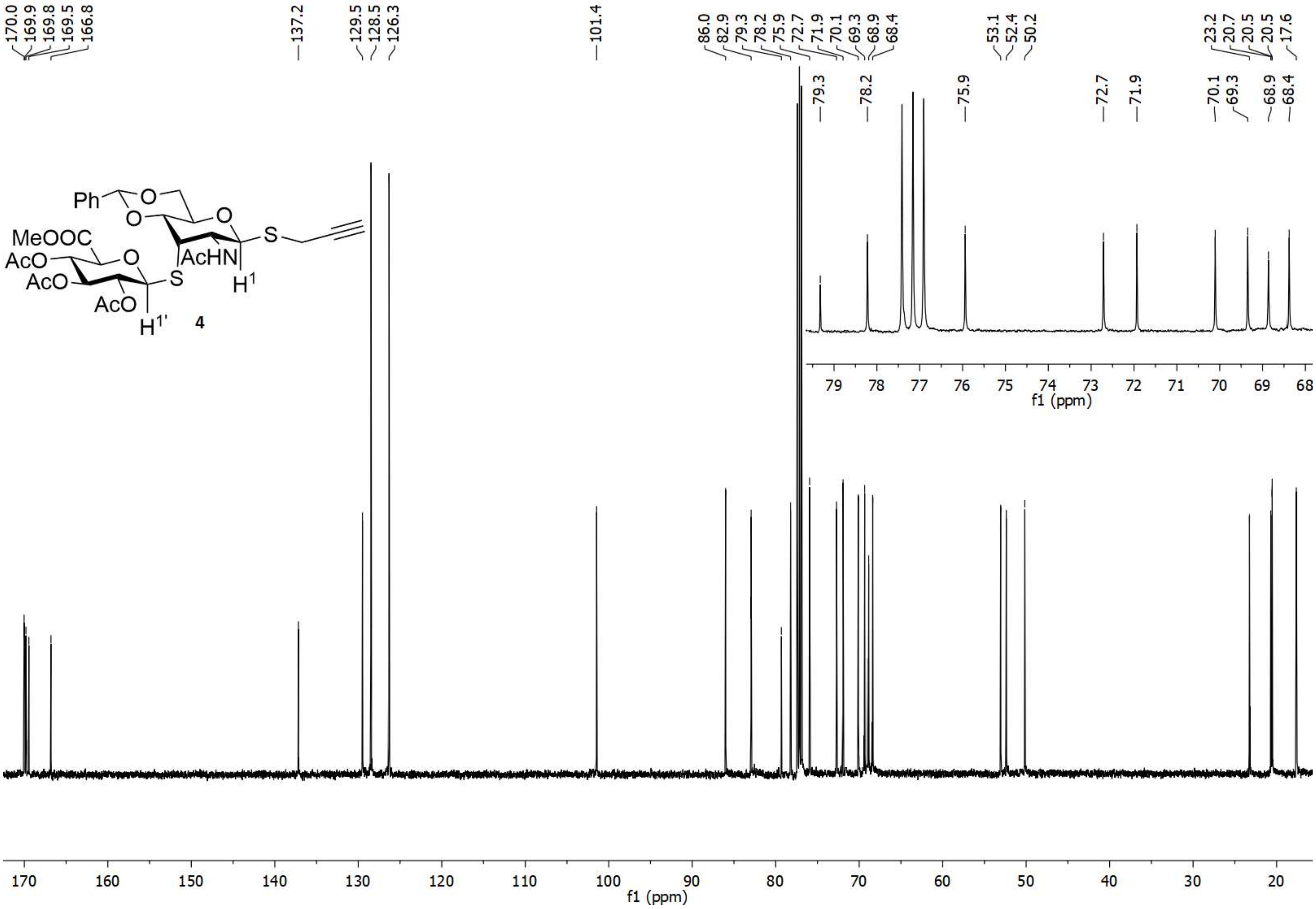
170.0
169.9
169.8
169.5
166.8
137.2
129.5
128.5
126.3
101.4
86.0
82.9
79.3
78.2
75.9
72.7
71.9
70.1
69.3
68.9
68.4
53.1
52.4
50.2
23.2
20.7
20.5
20.5
17.6
Ph
O
MeOOC
AcO
AcO
AcO
S
AcHN
H1
H1'
4
f1 (ppm)
f1 (ppm)

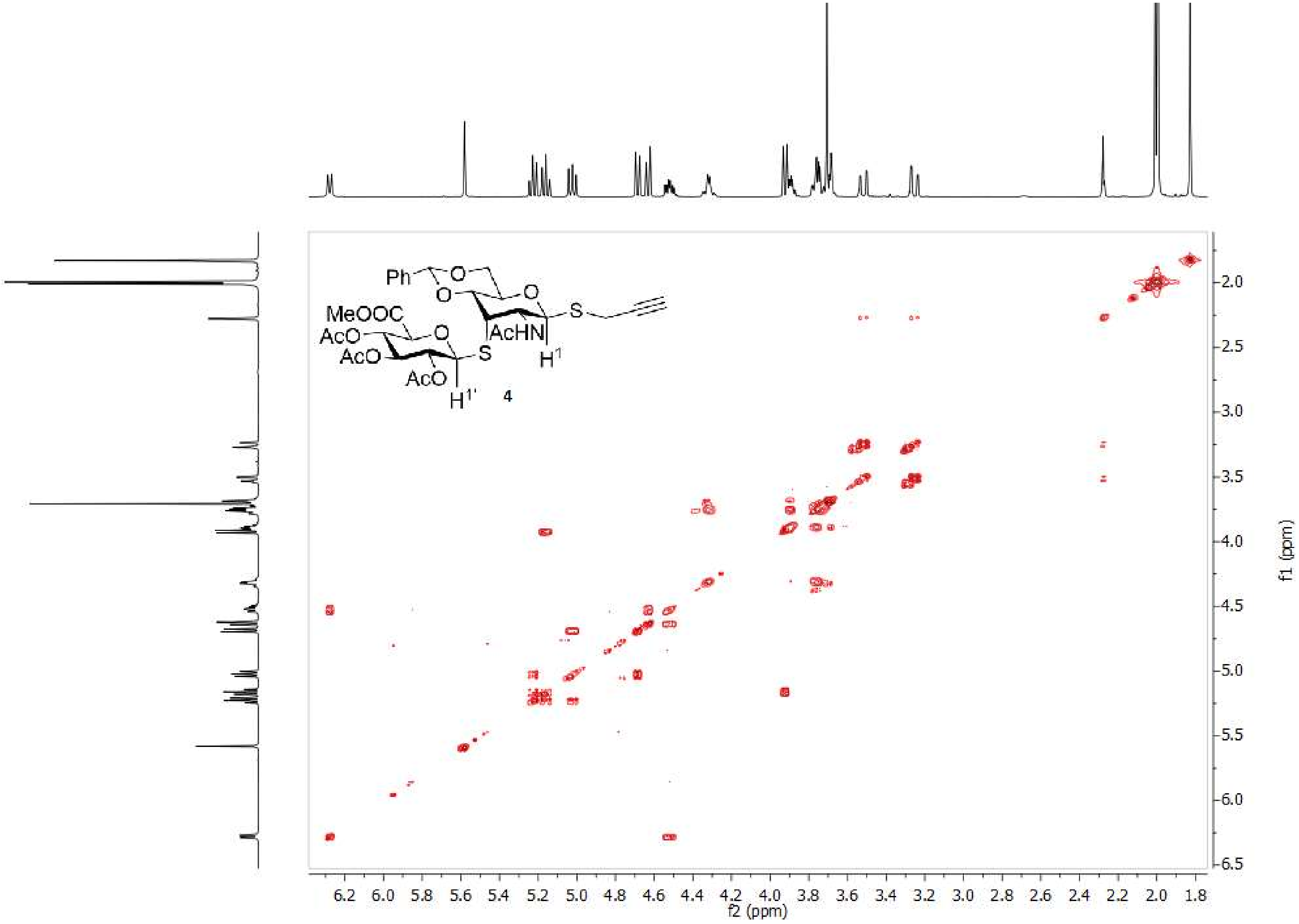

Ph
O
O
O
S
MeOOC
AcO
O
AcO
AcHN
S
H1
AcO
H1'
4
f1 (ppm)
f2 (ppm)

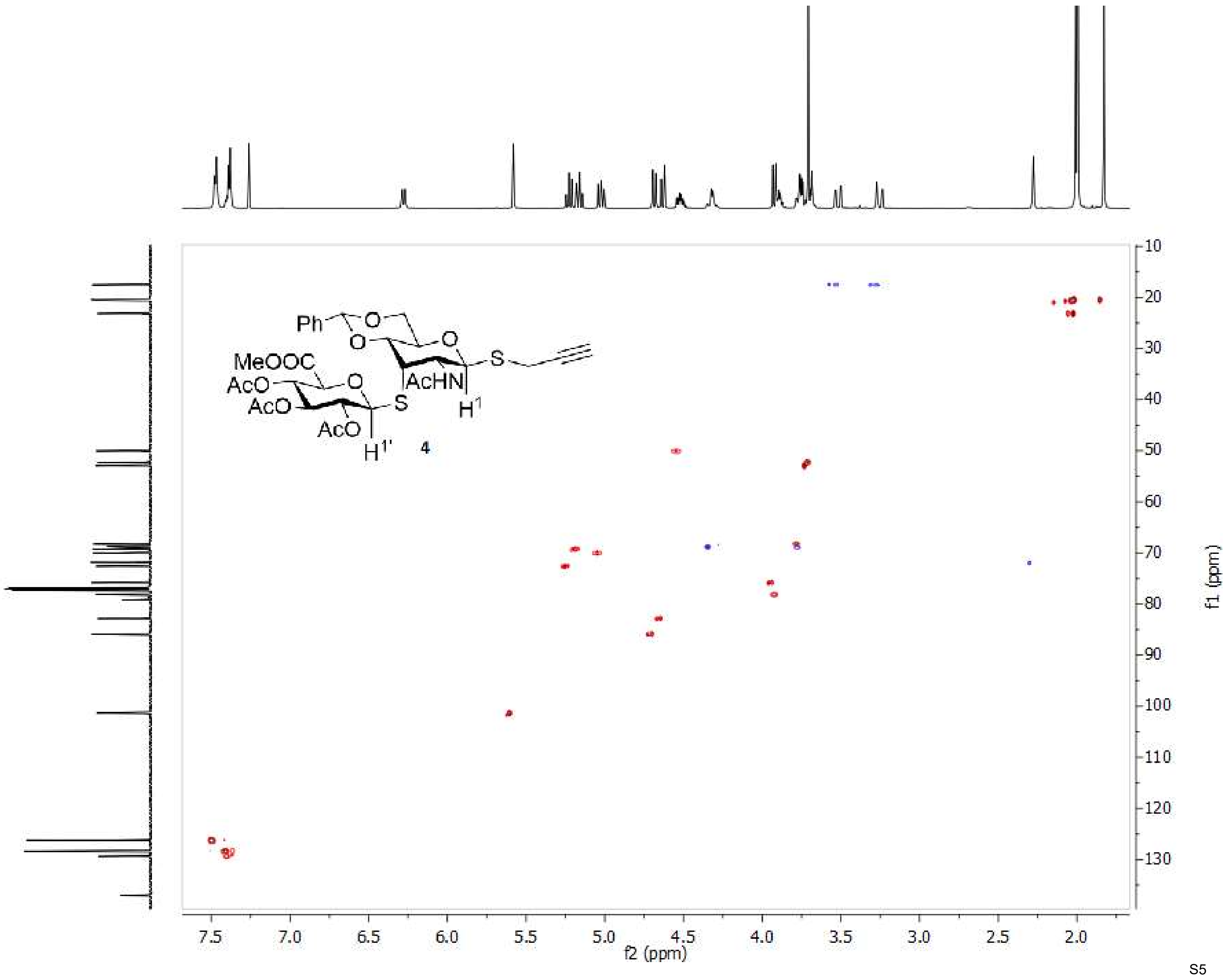
Ph
O
O
O
MeOOC
AcO
AcO
AcO
O
S
AcHN
S
H1
H1'
4
f2 (ppm)
f1 (ppm)

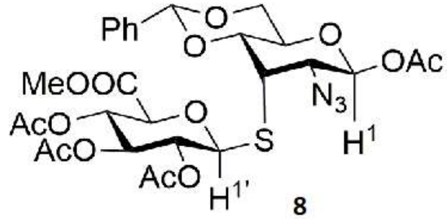
Ph
MeOOC
AcO
AcO
AcO
OAc
N_3
S
H^1
$H^{1'}$

8

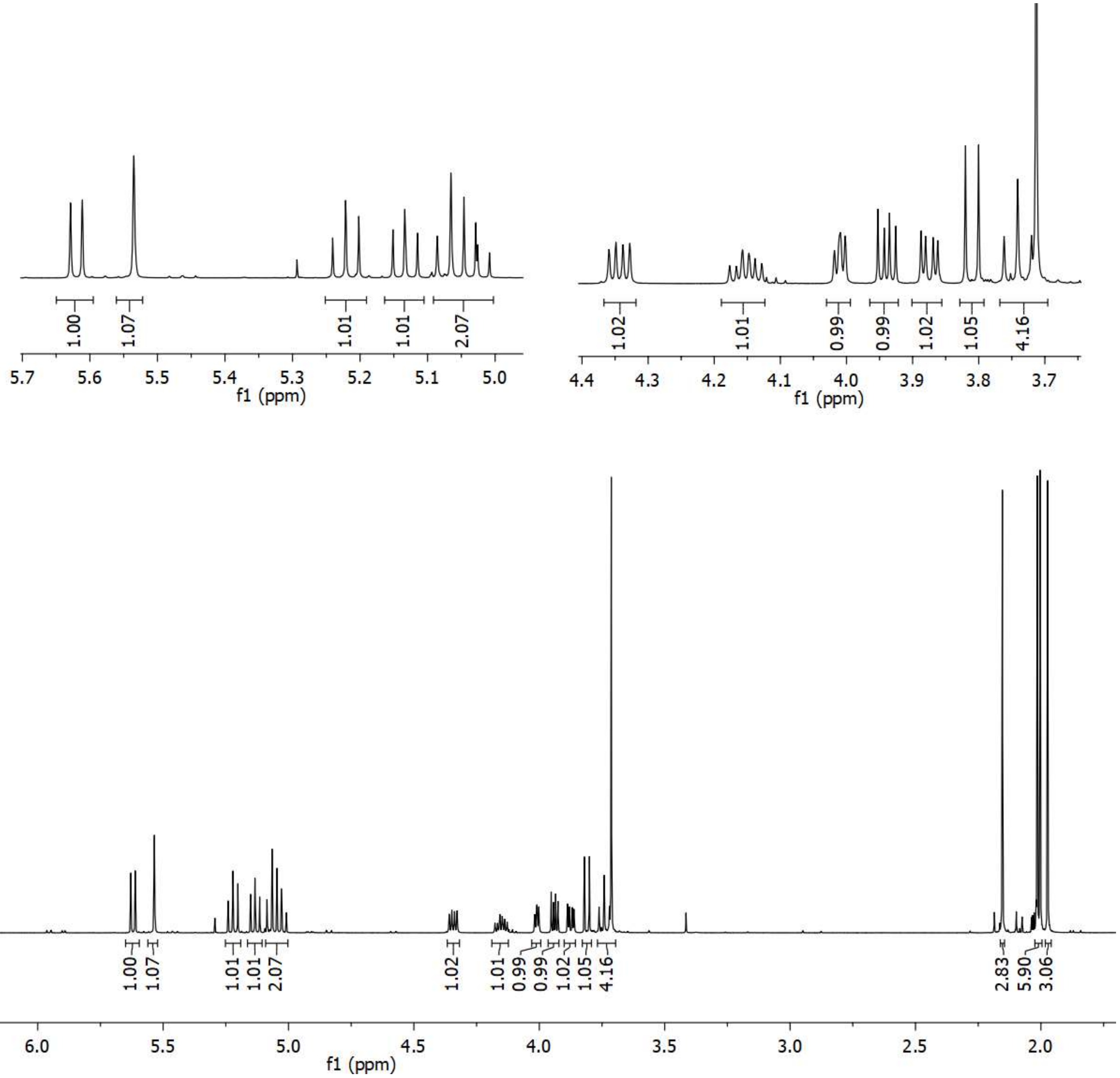
1.00
1.07
1.01
1.01
2.07
1.02
1.01
0.99
0.99
1.02
1.05
4.16
4.94
2.83
5.90
3.06
f1 (ppm)

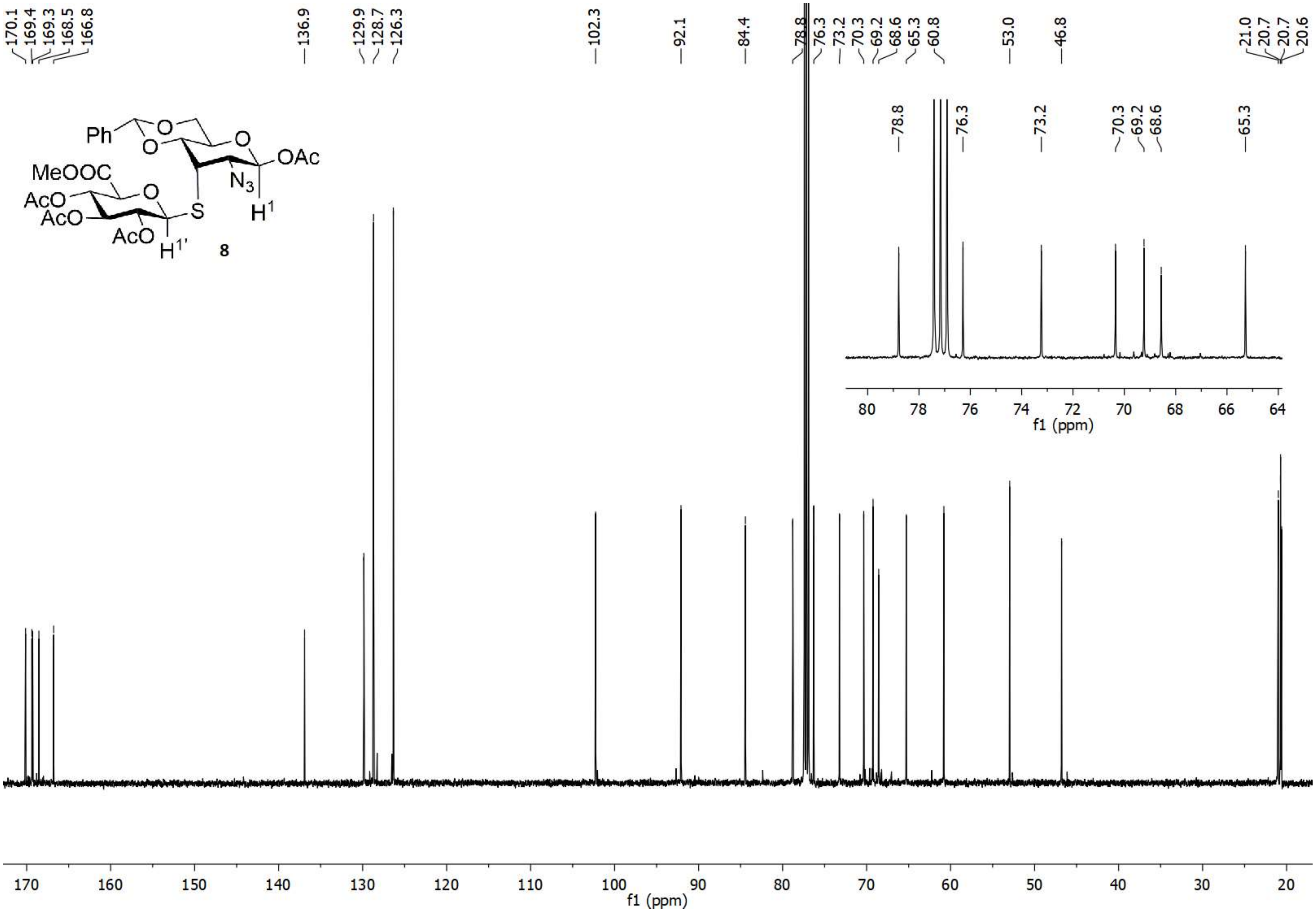
170.1
169.4
169.3
168.5
166.8
136.9
129.9
128.7
126.3
102.3
92.1
84.4
78.8
76.3
73.2
70.3
69.2
68.6
65.3
60.8
53.0
46.8
21.0
20.7
20.7
20.6
Ph
O
O
O
OAc
MeOOC
N3
AcO
AcO
S
H1
AcO
H1'
8
f1 (ppm)

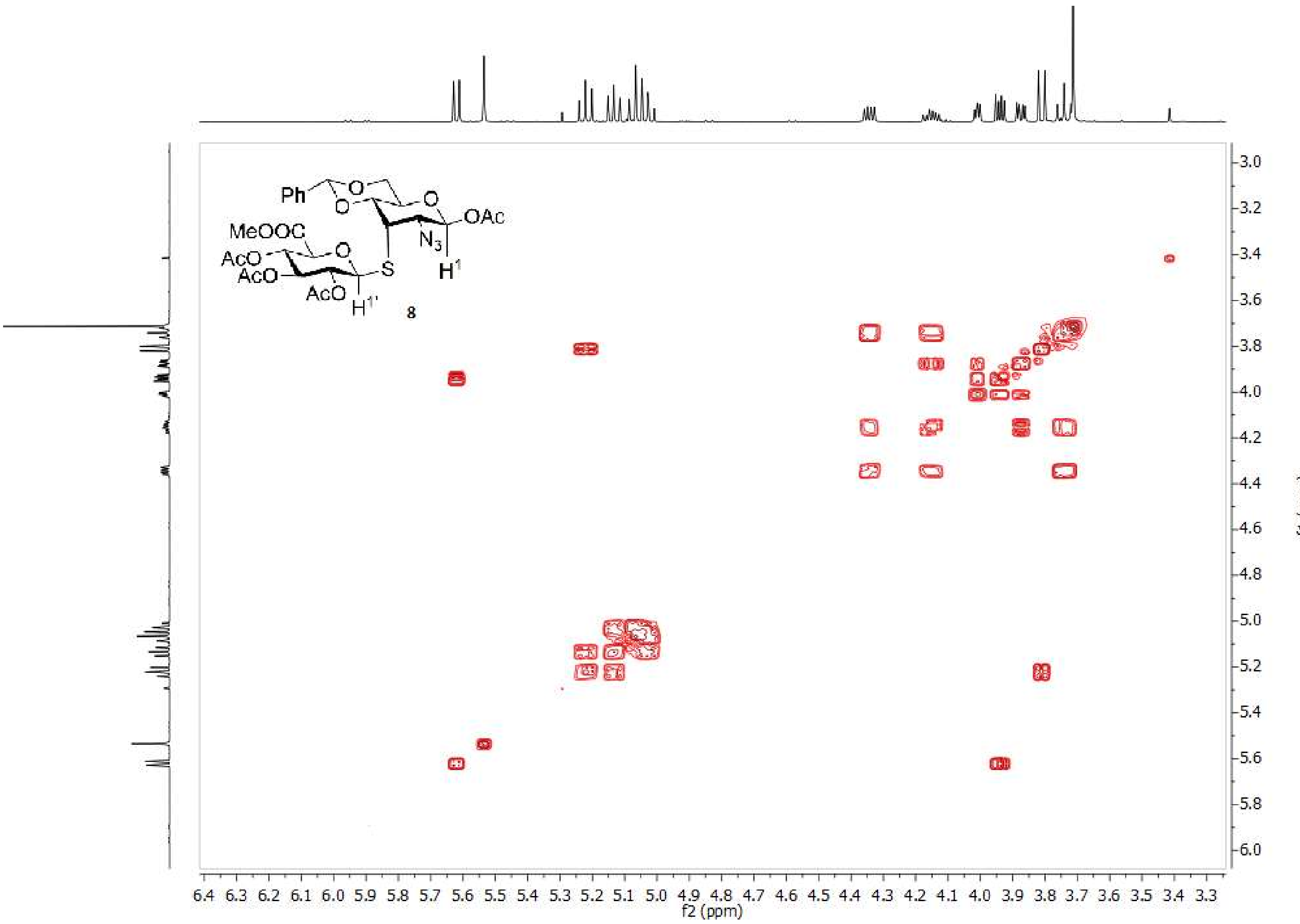
Ph
O
O
O
OAc
MeOOC
N3
AcO
O
AcO
S
H1
AcO
H1'
8
f2 (ppm)
f1 (ppm)

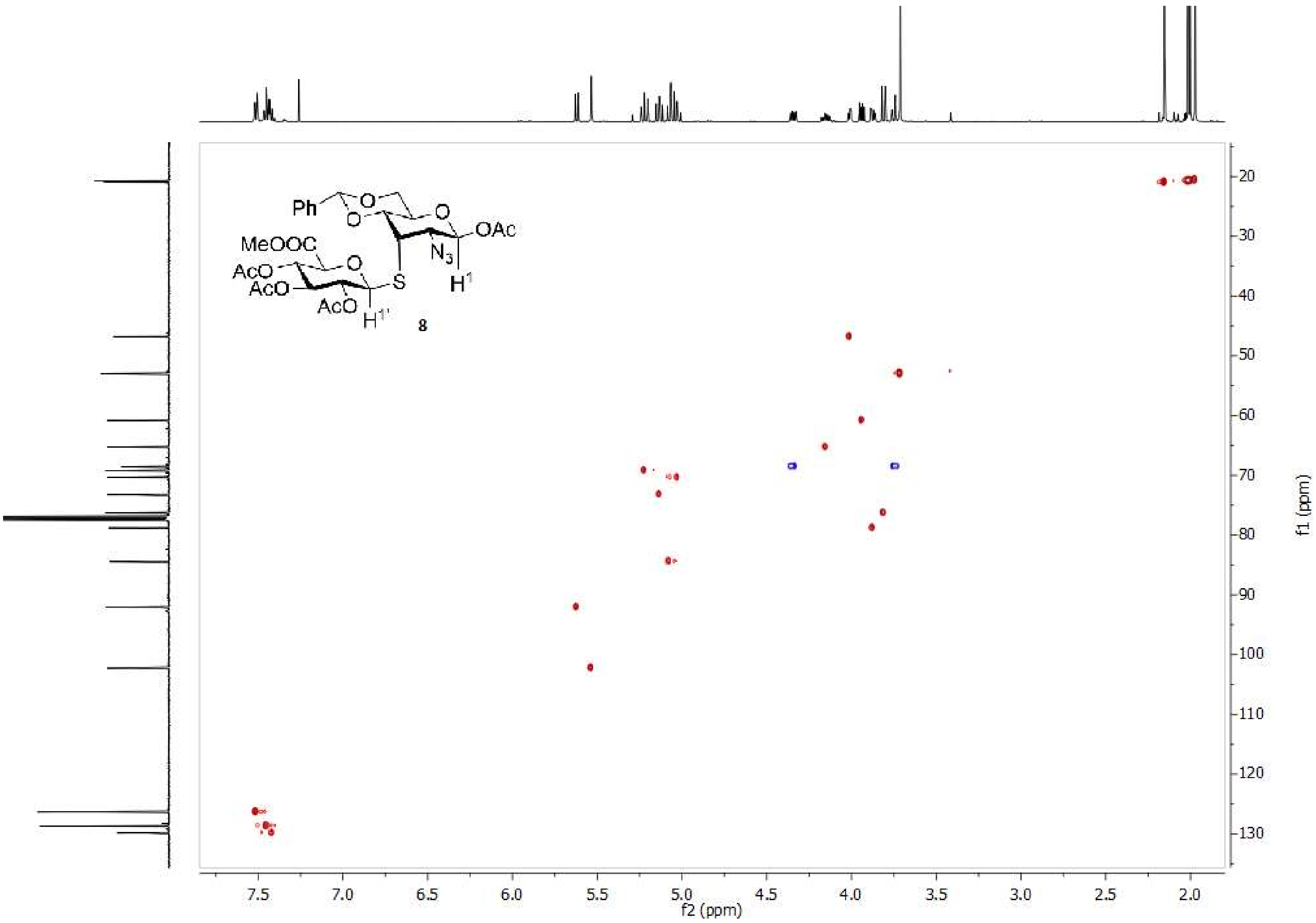
Ph
O
O
O
OAc
MeOOC
AcO
AcO
AcO
O
S
N_3
H^1
$H^{1'}$
8
f2 (ppm)
f1 (ppm)
7.5
7.0
6.5
6.0
5.5
5.0
4.5
4.0
3.5
3.0
2.5
2.0
20
30
40
50
60
70
80
90
100
110
120
130

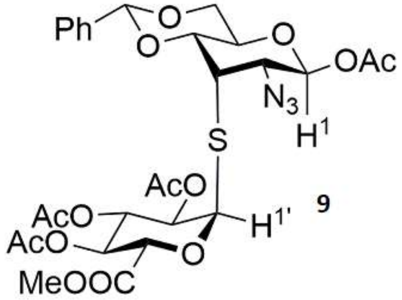

9

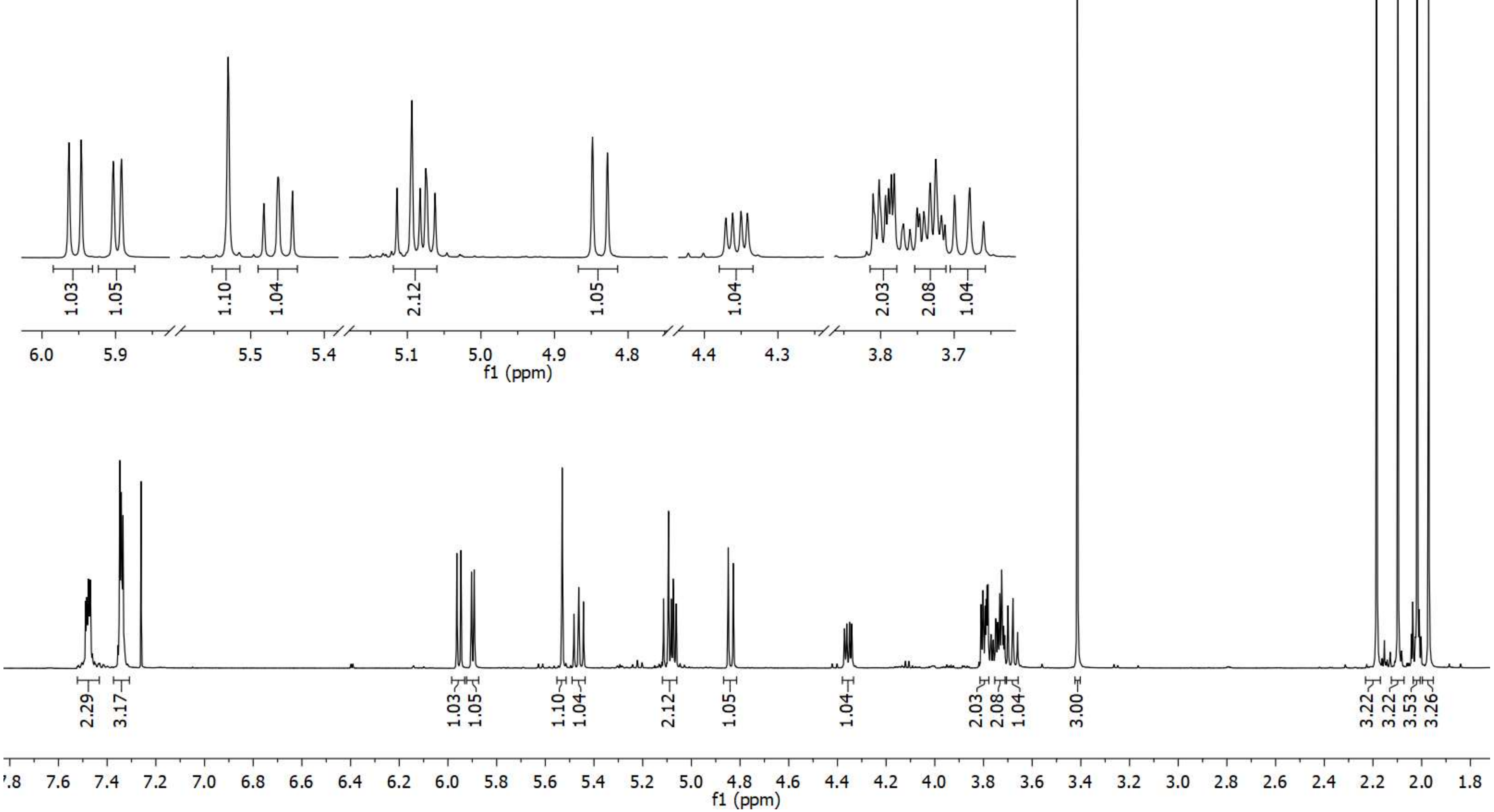

1.03
1.05
1.10
1.04
2.12
1.05
1.04
2.03
2.08
1.04
6.0
5.9
5.5
5.4
5.1
5.0
4.9
4.8
4.4
4.3
3.8
3.7
f1 (ppm)
2.29
3.17
1.03
1.05
1.10
1.04
2.12
1.05
1.04
2.03
2.08
1.04
3.00
3.22
3.22
3.53
3.26
7.6
7.4
7.2
7.0
6.8
6.6
6.4
6.2
6.0
5.8
5.6
5.4
5.2
5.0
4.8
4.6
4.4
4.2
4.0
3.8
3.6
3.4
3.2
3.0
2.8
2.6
2.4
2.2
2.0
1.8
f1 (ppm)

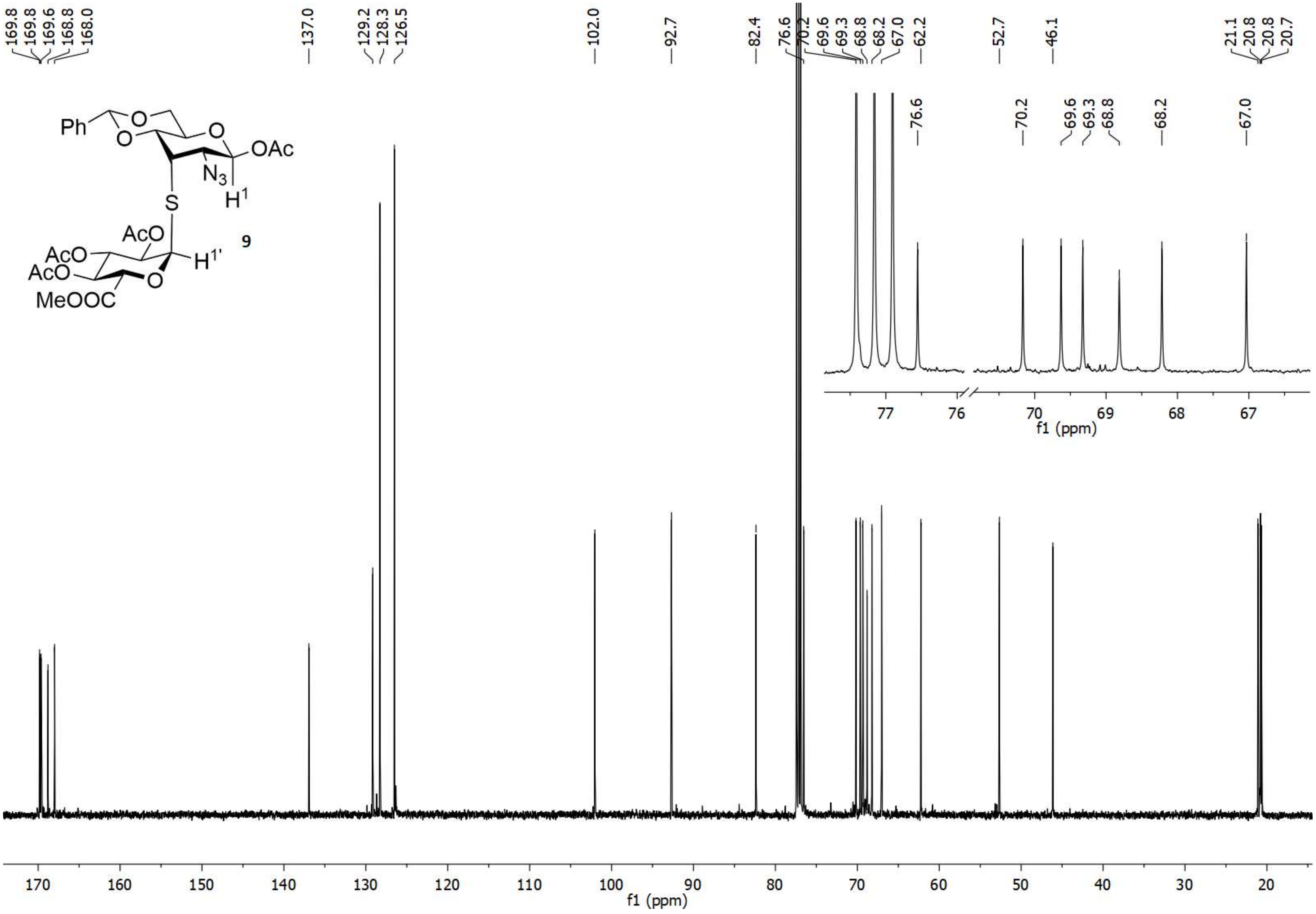
169.8
169.8
169.6
168.8
168.0
137.0
129.2
128.3
126.5
102.0
92.7
82.4
76.6
70.2
69.6
69.3
68.8
68.2
67.0
62.2
52.7
46.1
21.1
20.8
20.8
20.7
Ph
O
OAc
N3
H1
S
AcO
H1'
MeOOC
9
f1 (ppm)

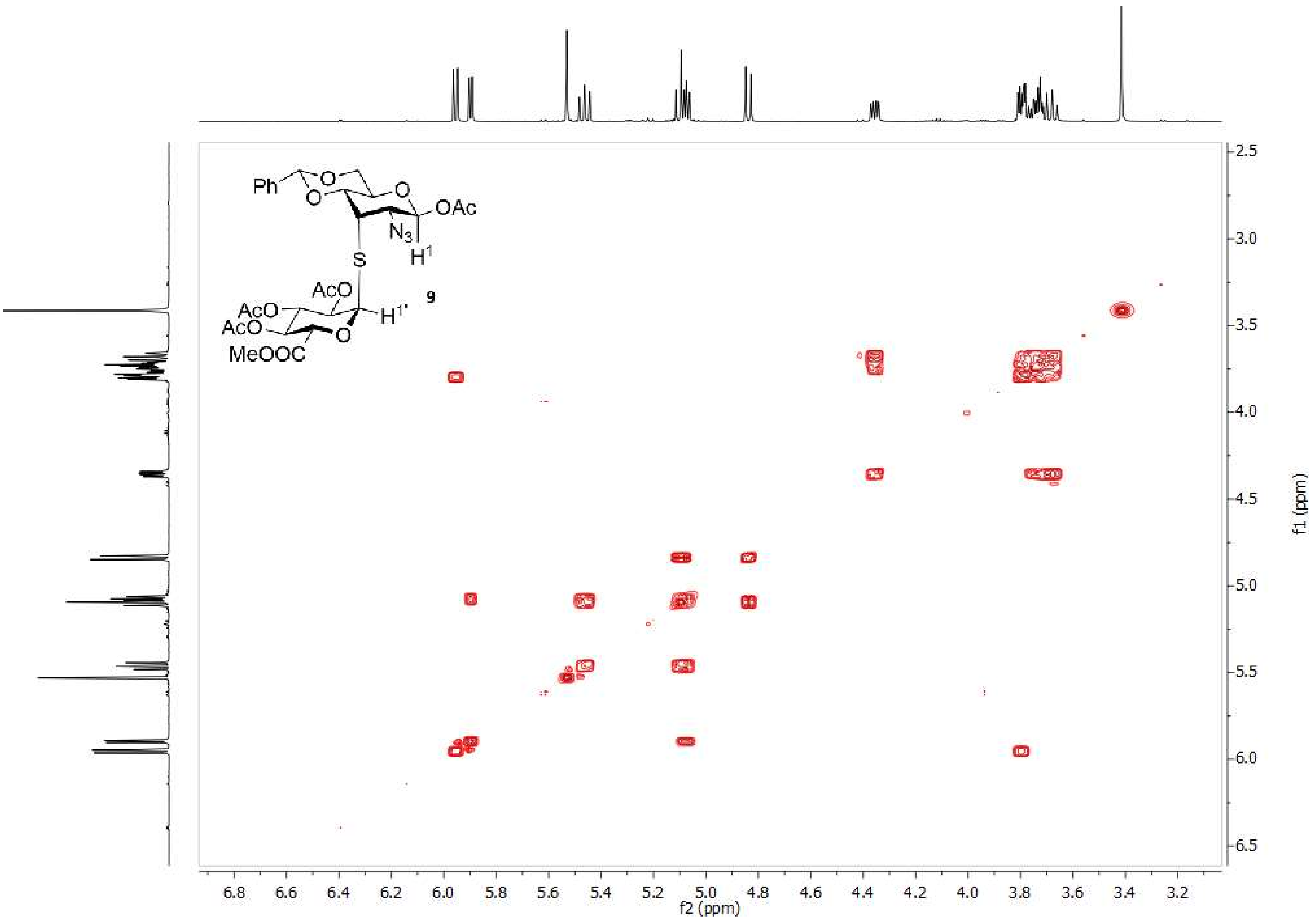
Ph
O
O
O
OAc
N3
H1
S
AcO
AcO
AcO
H1'
O
MeOOC
9
f2 (ppm)
f1 (ppm)

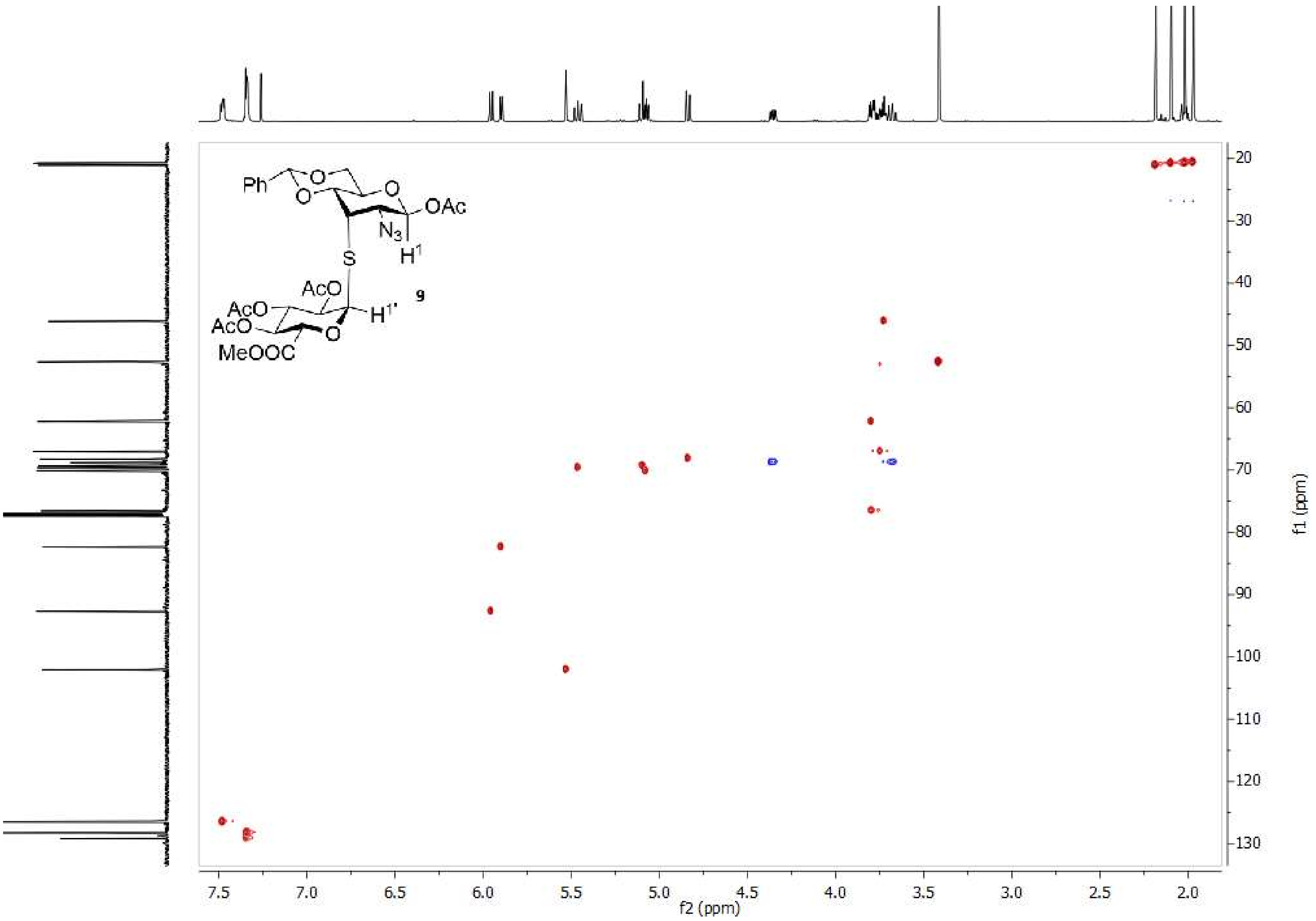
Ph
O
O
O
OAc
N3
H1
S
AcO
AcO
AcO
H1'
O
MeOOC
9
f2 (ppm)
f1 (ppm)

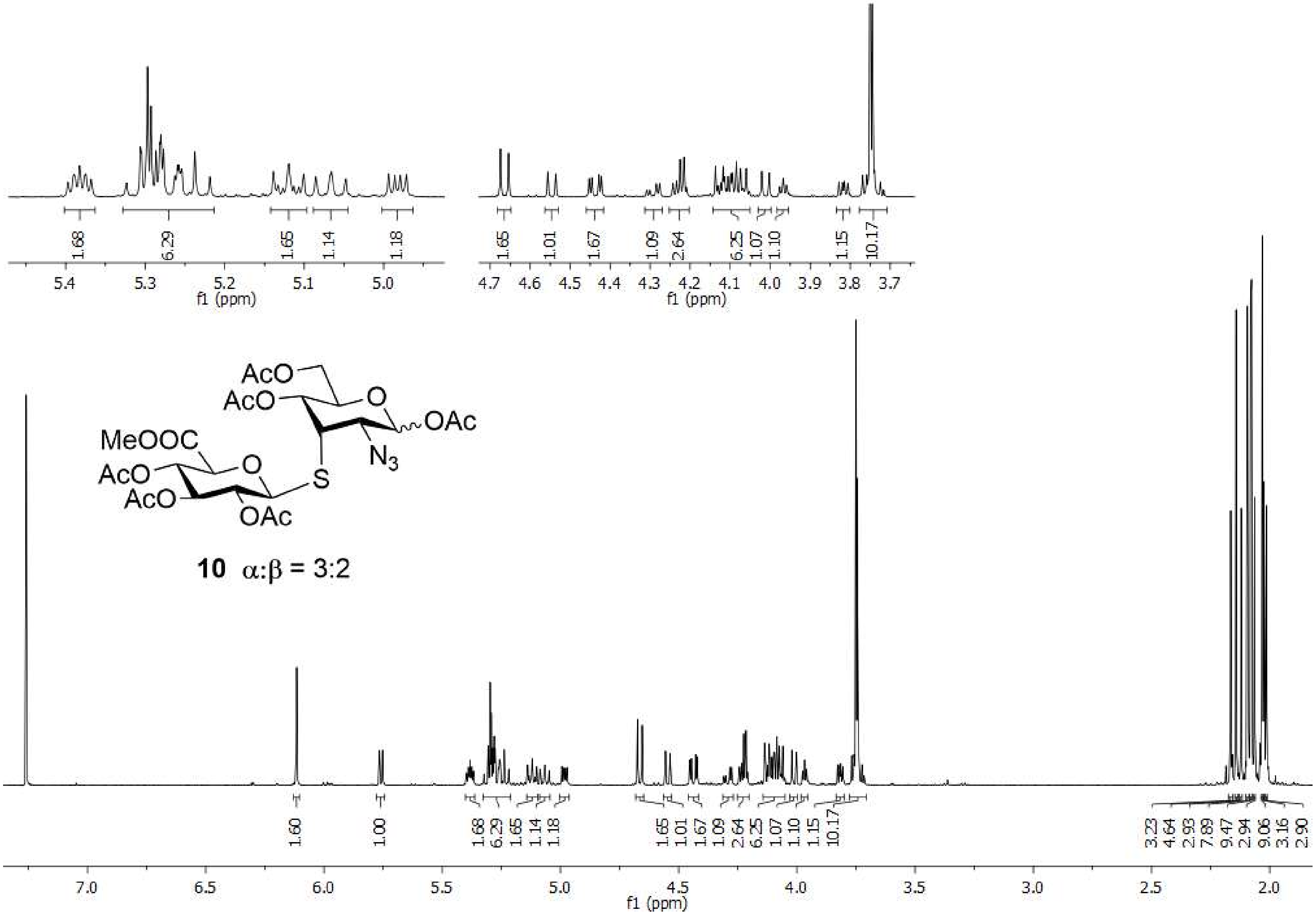
AcO
AcO
O
OAc
MeOOC
N3
AcO
O
S
AcO
OAc
10 α:β = 3:2
1.68
6.29
1.65
1.14
1.18
5.4
5.3
5.2
5.1
5.0
f1 (ppm)
1.65
1.01
1.67
1.09
2.64
6.25
1.07
1.10
1.15
10.17
4.7
4.6
4.5
4.4
4.3
4.2
4.1
4.0
3.9
3.8
3.7
f1 (ppm)
1.60
1.00
3.23
4.64
2.93
7.89
9.47
2.94
9.06
3.16
2.90
7.0
6.5
6.0
5.5
5.0
4.5
4.0
3.5
3.0
2.5
2.0
f1 (ppm)

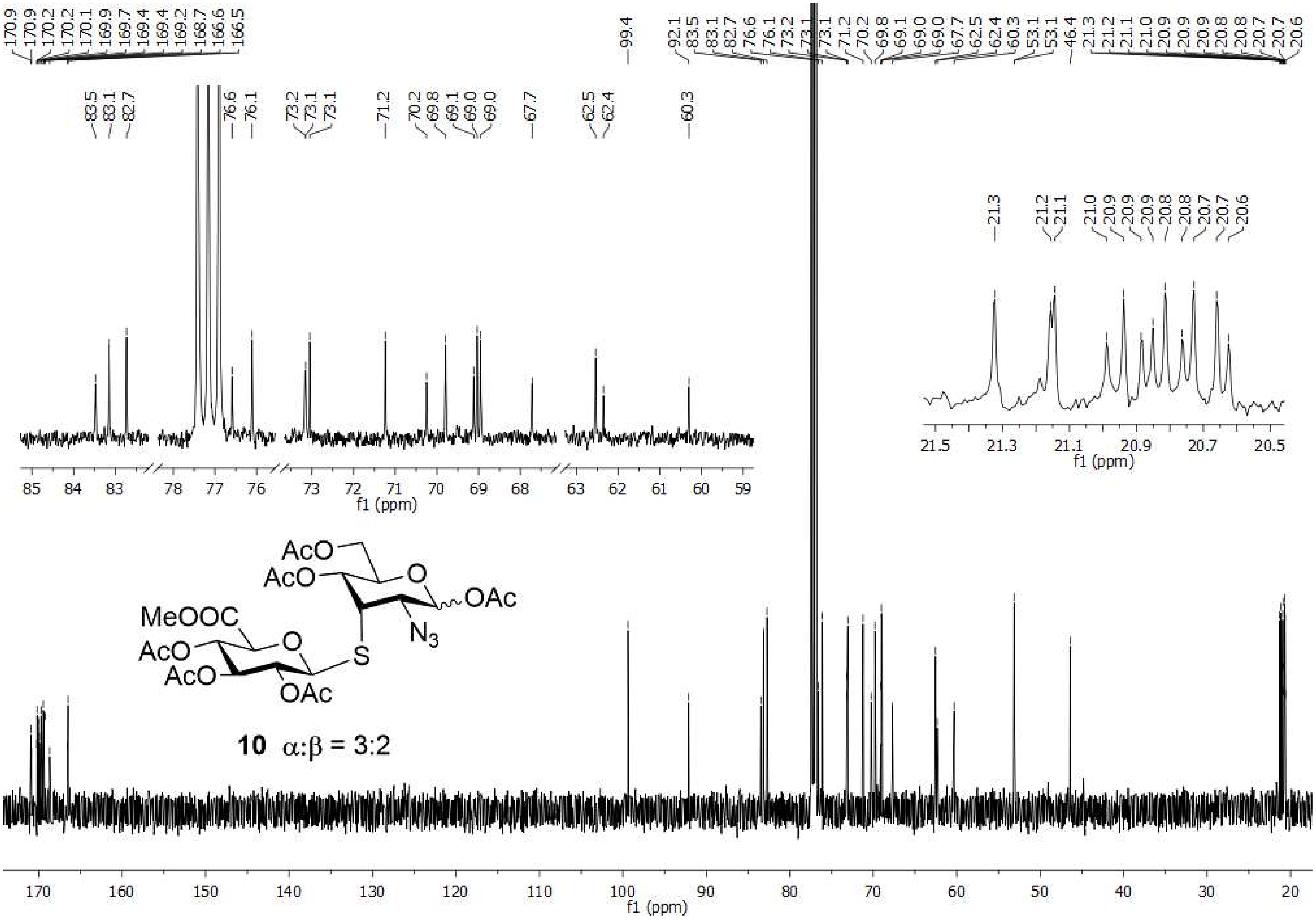
170.9 170.9 170.2 170.2 170.1 169.9 169.7 169.4 169.4 169.2 168.7 166.6 166.5
99.4
92.1 83.5 83.1 82.7 76.6 76.1 73.2 73.1 73.1 71.2 70.2 69.8 69.1 69.0 69.0 67.7 62.5 62.4 60.3 53.1 53.1 46.4 21.3 21.2 21.1 21.0 20.9 20.9 20.9 20.8 20.8 20.7 20.7 20.6
83.5 83.1 82.7 76.6 76.1 73.2 73.1 73.1 71.2 70.2 69.8 69.1 69.0 69.0 67.7 62.5 62.4 60.3
85 84 83 78 77 76 73 72 71 70 69 68 63 62 61 60 59
f1 (ppm)
21.3 21.2 21.1 21.0 20.9 20.9 20.9 20.8 20.8 20.7 20.7 20.6
21.5 21.3 21.1 20.9 20.7 20.5
f1 (ppm)
170 160 150 140 130 120 110 100 90 80 70 60 50 40 30 20
f1 (ppm)
AcO
AcO
O
OAc
MeOOC
O
AcO
AcO
S
N3
OAc

10 α:β = 3:2

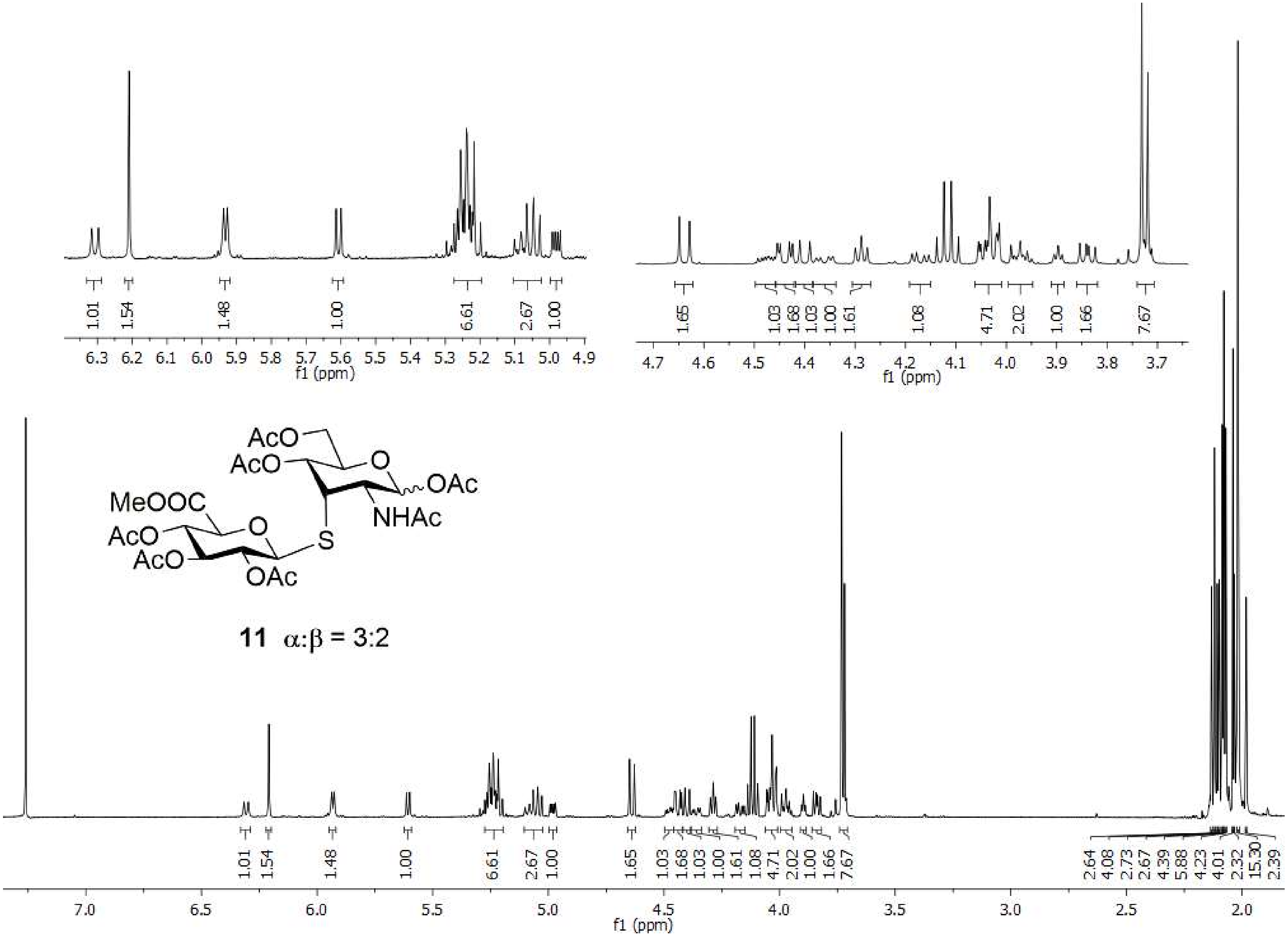
AcO
AcO
O
OAc
MeOOC
O
S
NHAc
AcO
AcO
OAc
11 α:β = 3:2
1.01 1.54 1.48 1.00 6.61 2.67 1.00
6.3 6.2 6.1 6.0 5.9 5.8 5.7 5.6 5.5 5.4 5.3 5.2 5.1 5.0 4.9
f1 (ppm)
1.65 1.03 1.68 1.03 1.00 1.61 1.08 4.71 2.02 1.00 1.66 7.67
4.7 4.6 4.5 4.4 4.3 4.2 4.1 4.0 3.9 3.8 3.7
f1 (ppm)
1.01 1.54 1.48 1.00 6.61 2.67 1.00 1.65 1.03 1.68 1.03 1.00 1.61 1.08 4.71 2.02 1.00 1.66 7.67 2.64 4.08 2.73 2.67 4.39 5.88 4.23 4.01 2.32 15.30 2.39
7.0 6.5 6.0 5.5 5.0 4.5 4.0 3.5 3.0 2.5 2.0
f1 (ppm)

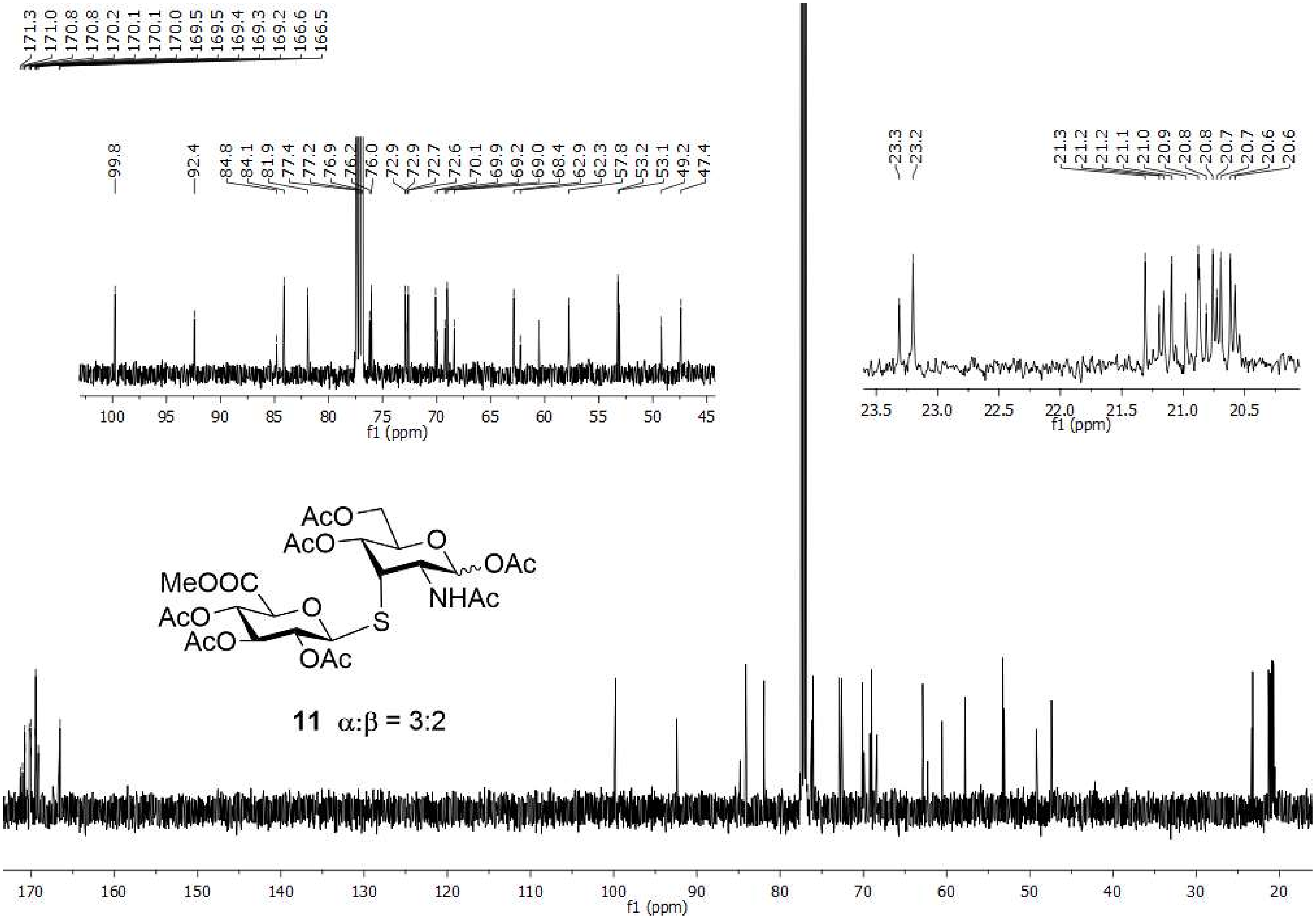

11 α:β = 3:2

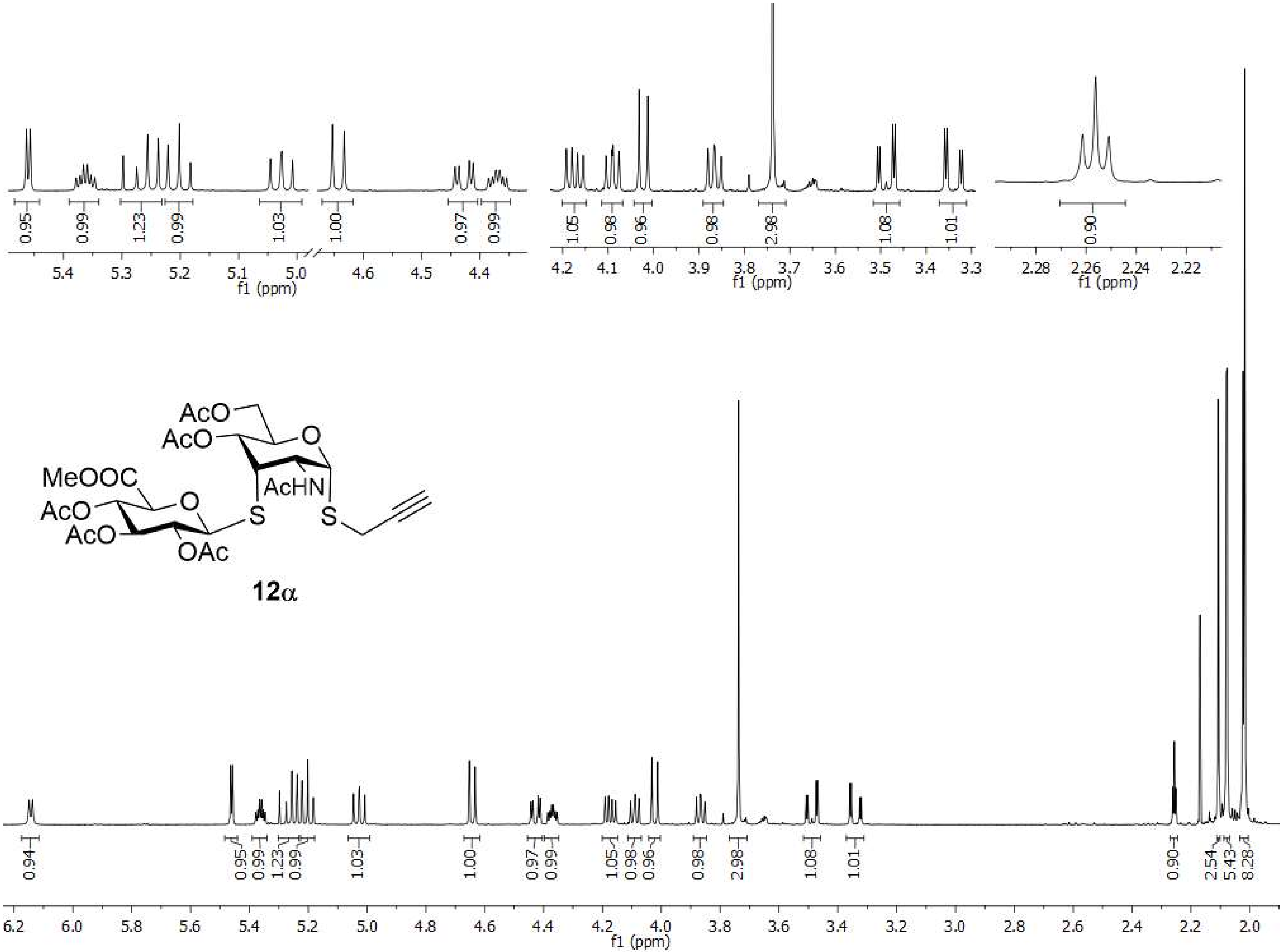
0.95 0.99 1.23 0.99 1.03 1.00 0.97 0.99
f1 (ppm)
1.05 0.98 0.96 0.98 2.98 1.08 1.01
f1 (ppm)
0.90
f1 (ppm)
0.94 0.95 0.99 1.23 0.99 1.03 1.00 0.97 0.99 1.05 0.98 0.96 0.98 2.98 1.08 1.01 0.90 2.54 5.43 8.28
f1 (ppm)
AcO
AcO
MeOOC
AcHN
AcO
AcO
S
S
OAc
O
O

12α

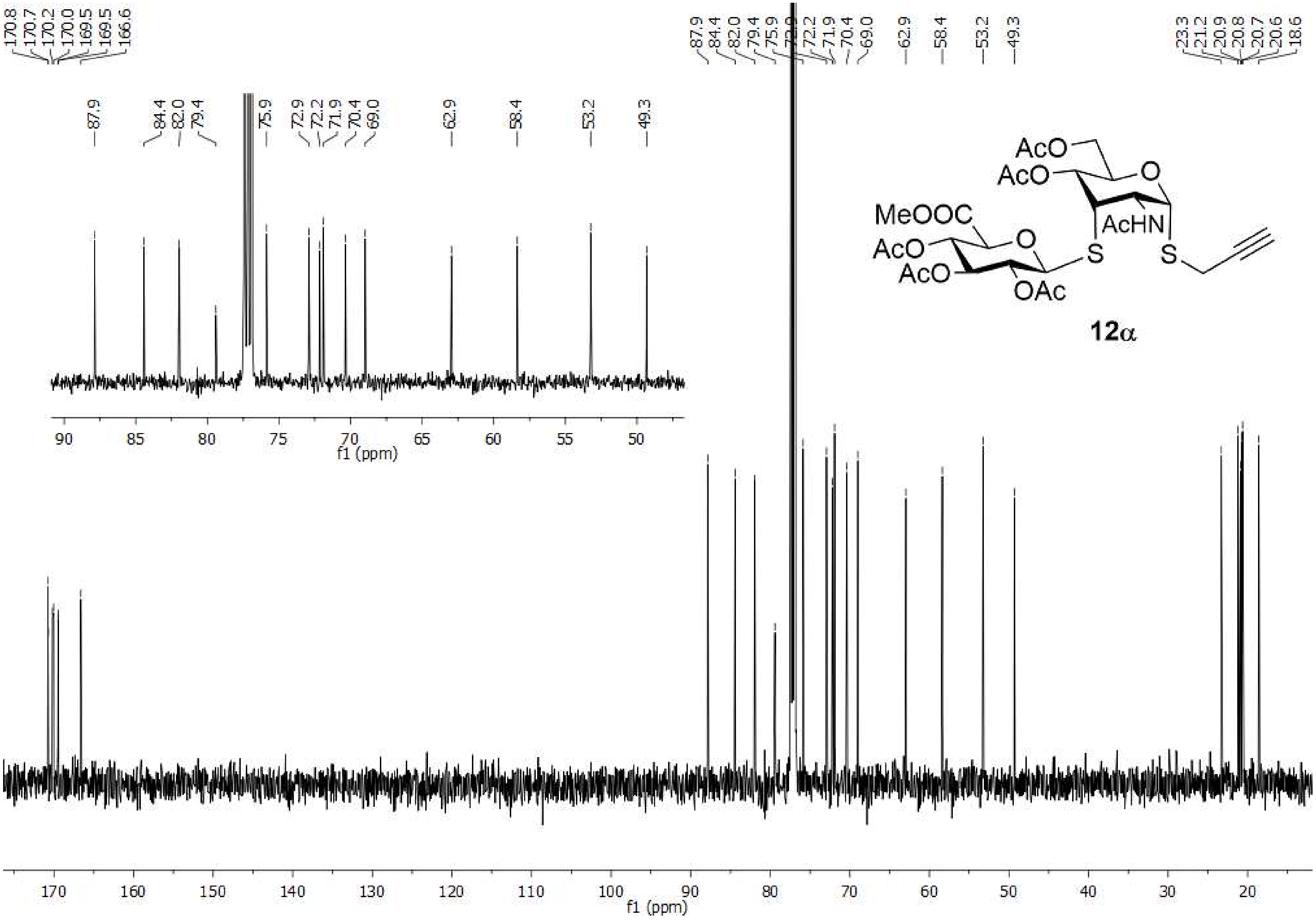
170.8
170.7
170.2
170.0
169.5
169.5
166.6
87.9
84.4
82.0
79.4
75.9
72.2
71.9
70.4
69.0
62.9
58.4
53.2
49.3
23.3
21.2
20.9
20.8
20.7
20.6
18.6
72.9
AcO
AcO
O
MeOOC
O
AcHN
AcO
AcO
S
S
OAc
12α
f1 (ppm)
90
85
80
75
70
65
60
55
50
170
160
150
140
130
120
110
100
40
30
20

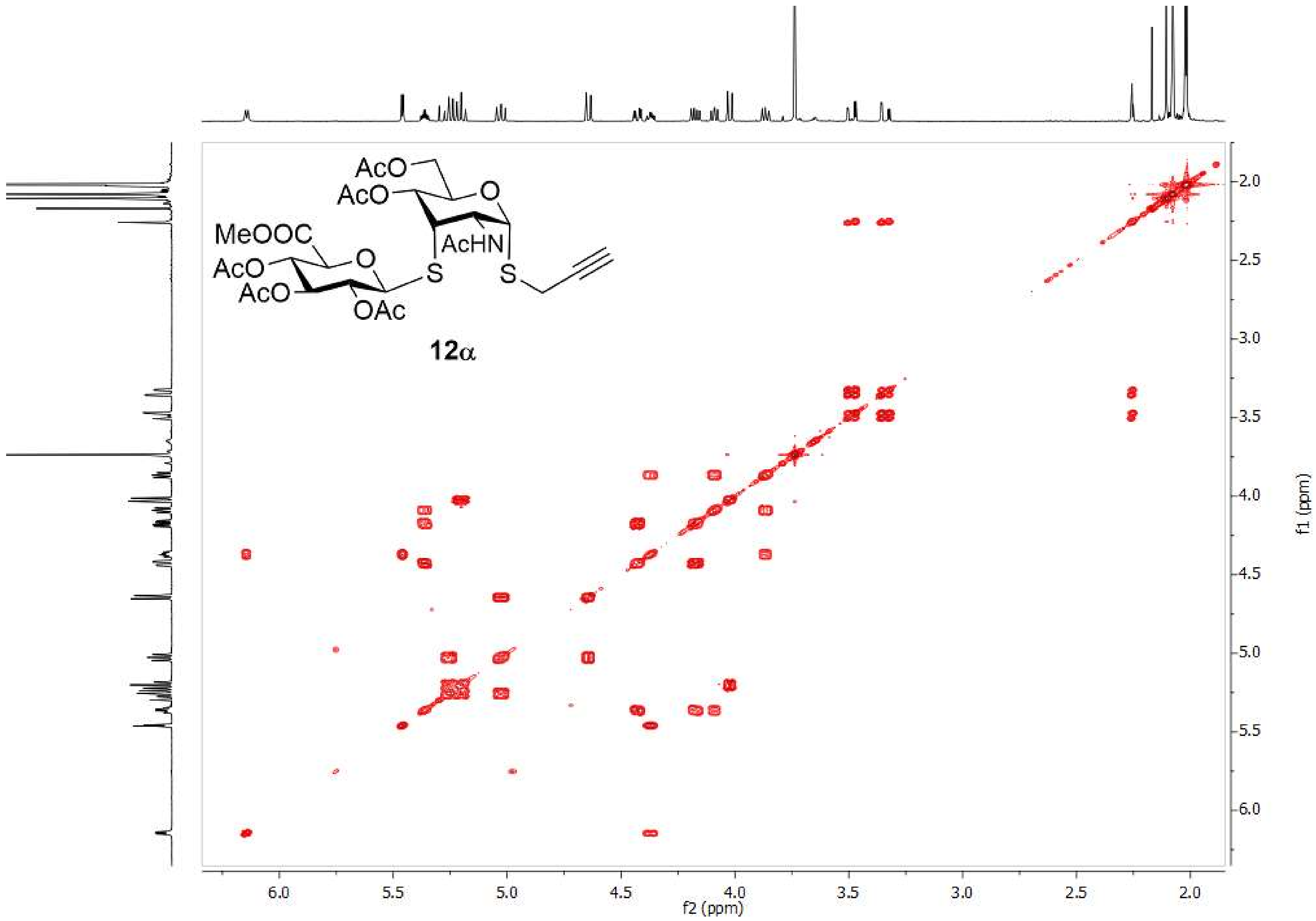
AcO
AcO
O
MeOOC
AcHN
AcO
O
S
S
AcO
OAc
12α
f2 (ppm)
f1 (ppm)

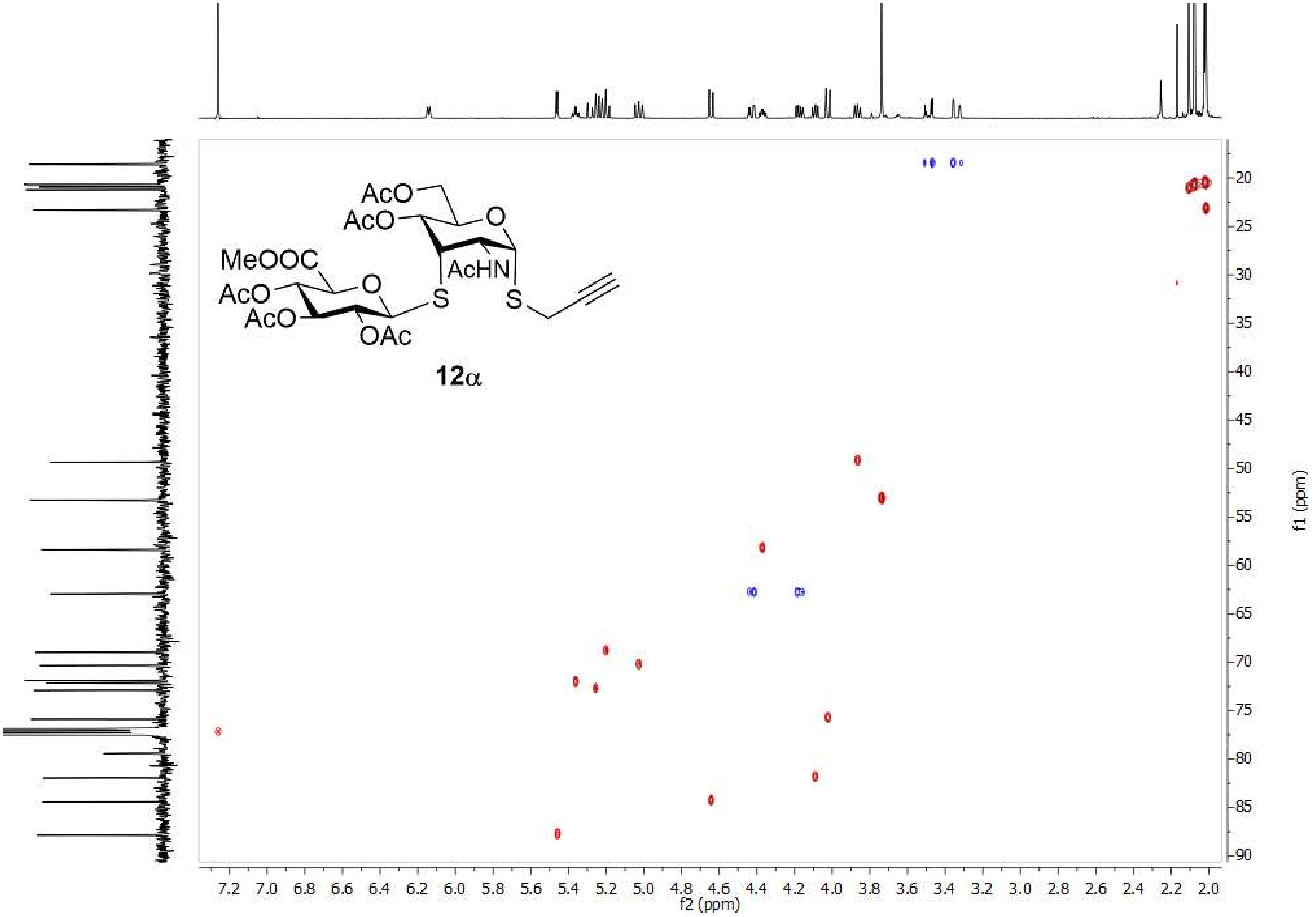
AcO
AcO
O
MeOOC
AcO
AcO
O
OAc
S
AcHN
S
12α
f1 (ppm)
f2 (ppm)

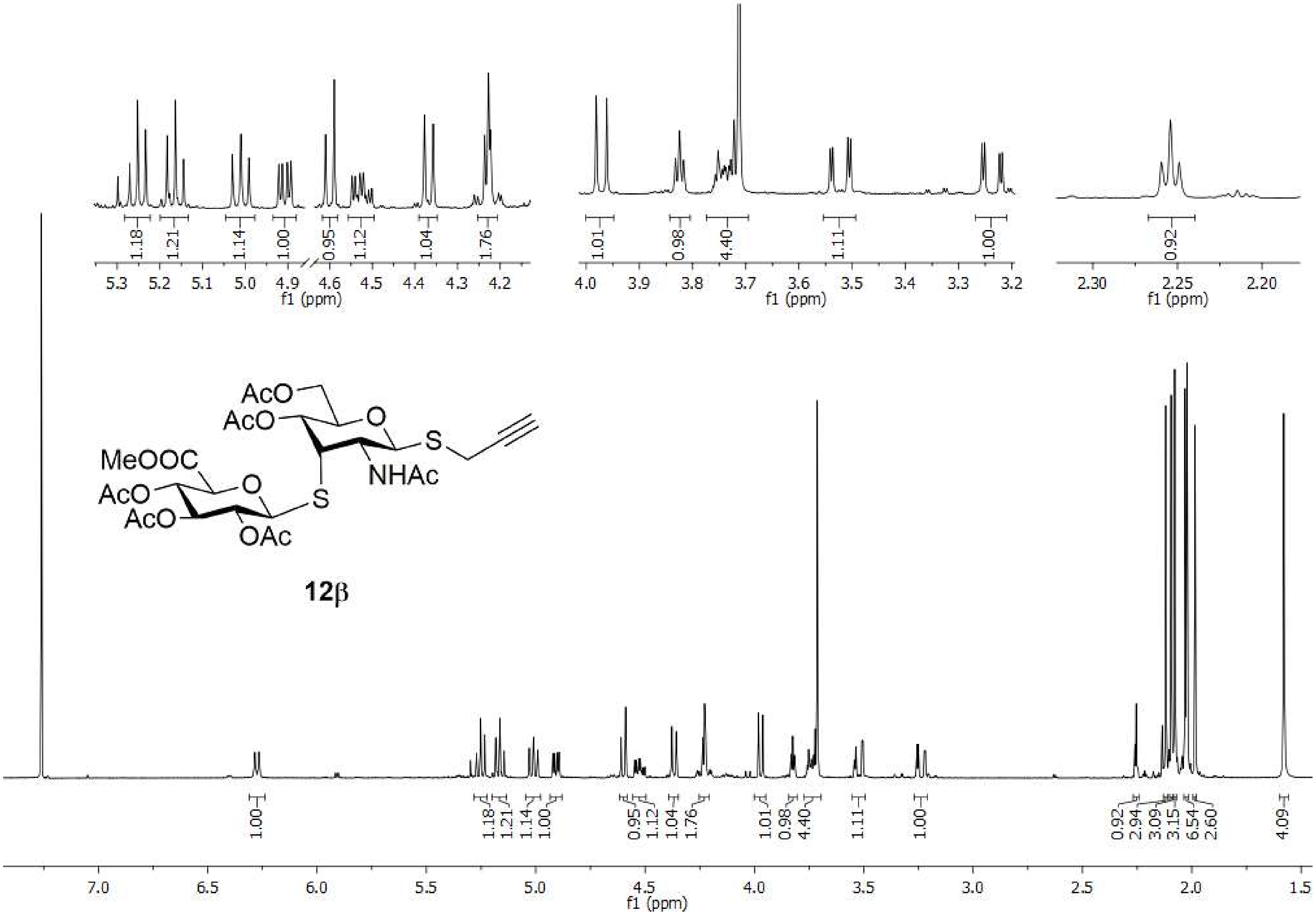

MeOOC
AcO
AcO
OAc
S
AcO
AcO
O
NHAc
S
f1 (ppm)

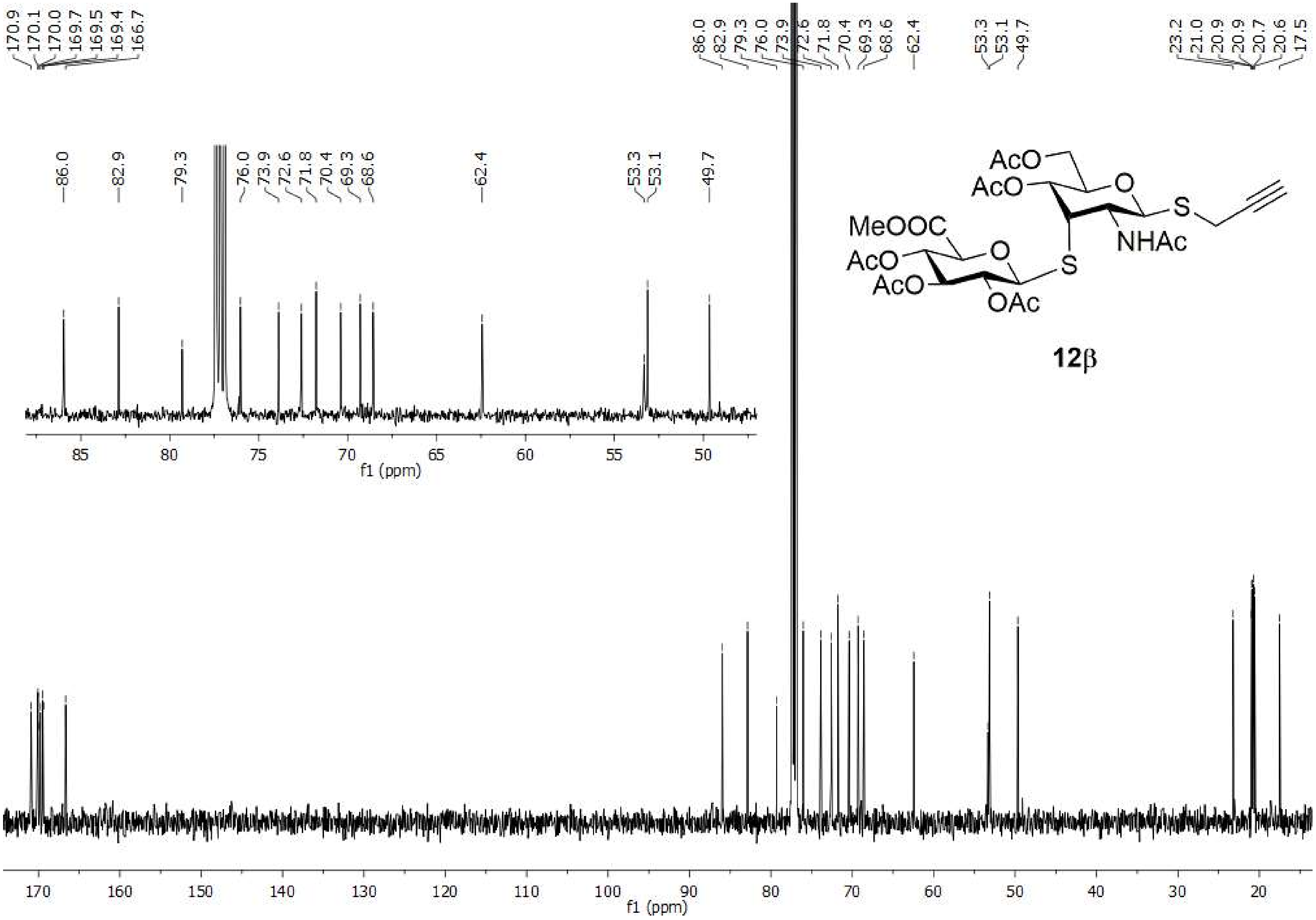
170.9
170.1
170.0
169.7
169.5
169.4
166.7
86.0
82.9
79.3
76.0
73.9
72.6
71.8
70.4
69.3
68.6
62.4
53.3
53.1
49.7
23.2
21.0
20.9
20.9
20.7
20.6
17.5
f1 (ppm)
f1 (ppm)
AcO
AcO
O
S
MeOOC
NHAc
AcO
O
AcO
S
OAc

12β

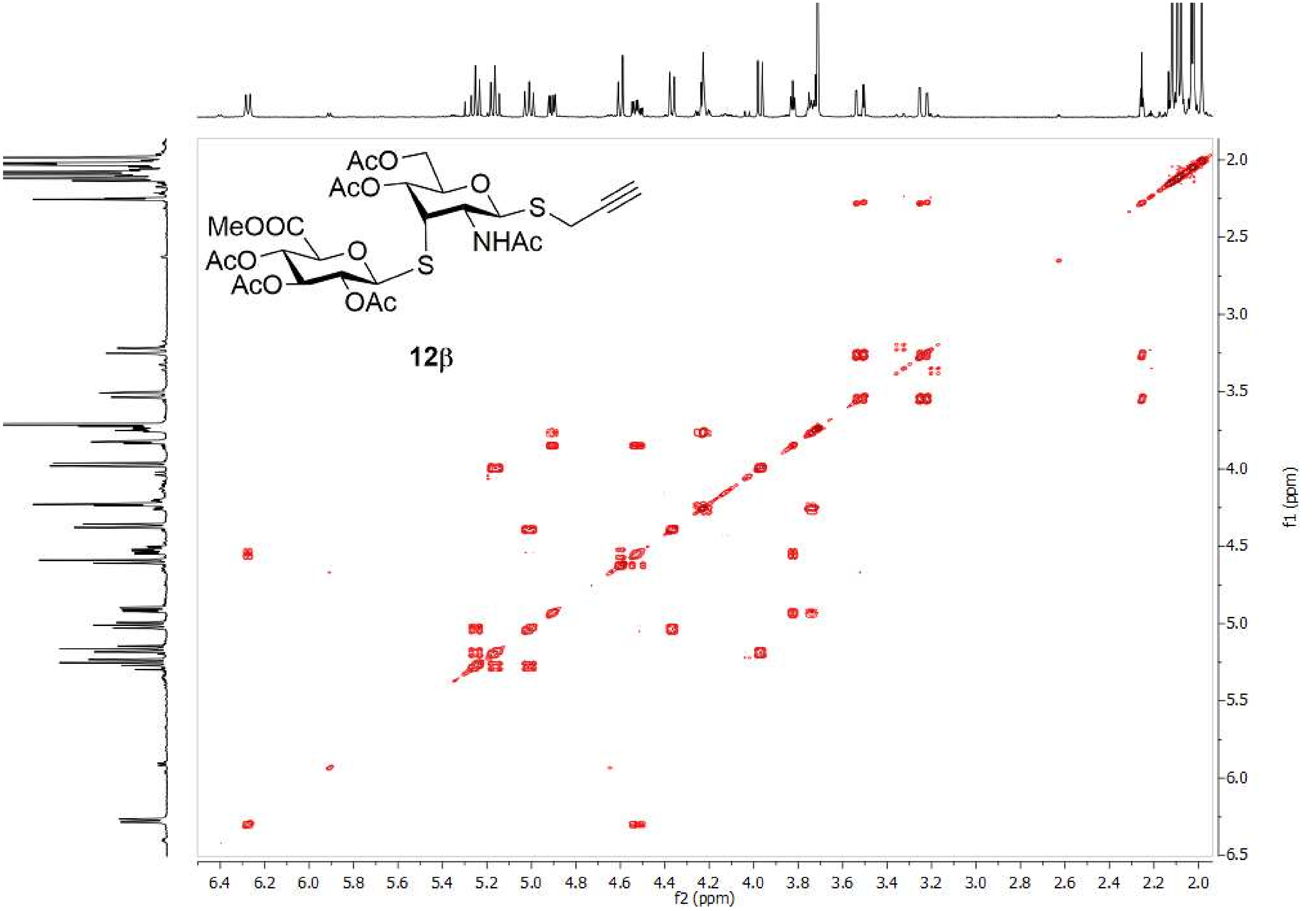
AcO
AcO
MeOOC
AcO
AcO
OAc
O
O
S
S
NHAc
12β
f2 (ppm)
f1 (ppm)

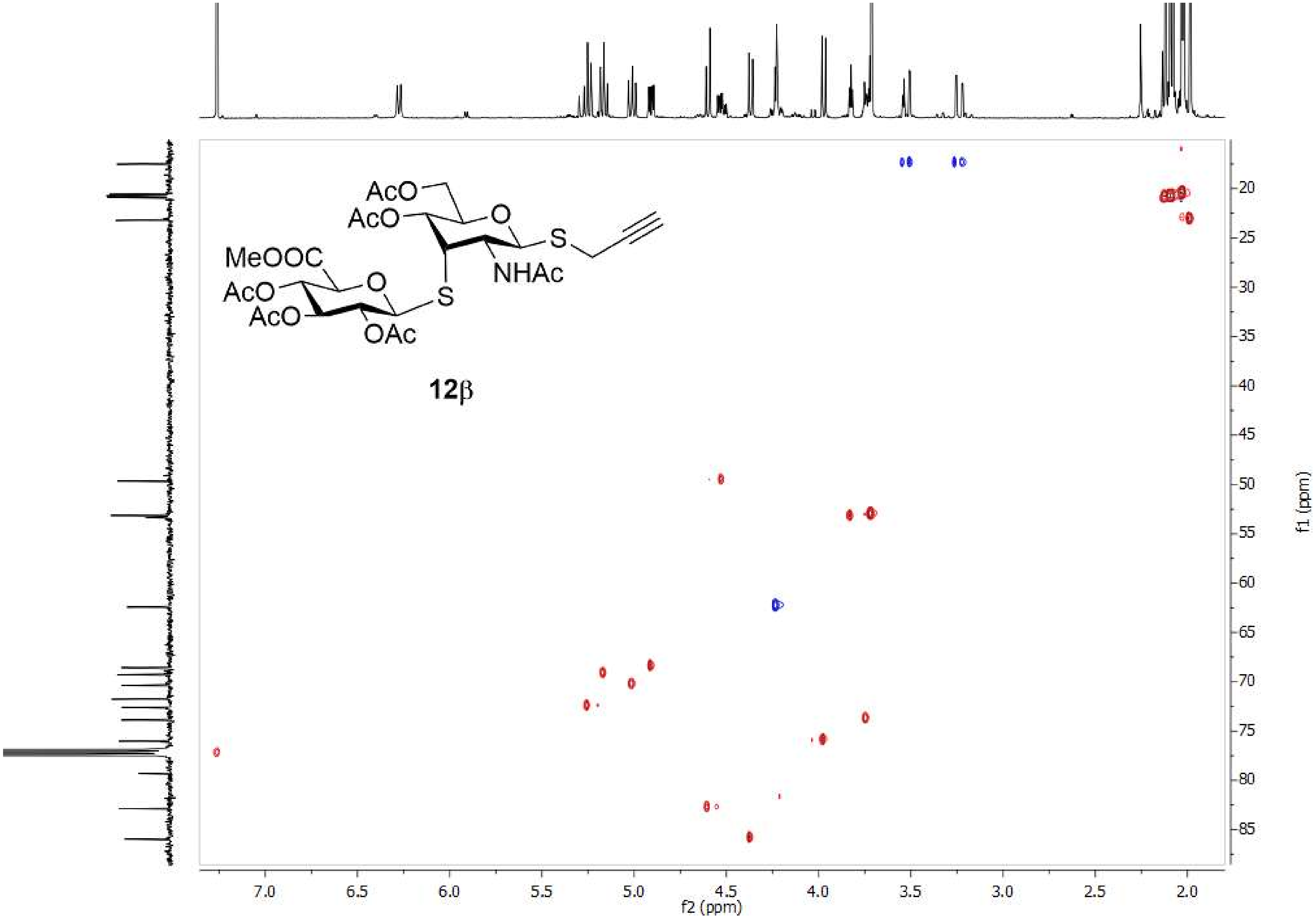

MeOOC
AcO
AcO
OAc
O
S
AcO
AcO
O
NHAc
S
12β
f2 (ppm)
f1 (ppm)
